# Advances in Wearable Thermoelectric Devices: Strategies for Enhancing Wearability and Performance

**DOI:** 10.1002/smsc.70402

**Published:** 2026-09-21

**Authors:** Yurim Lee, Hyein Ham, Siyeon Park, Hanbit Jang, Seungwan Hong, Jungmok Seo

**Affiliations:** ^1^ Department of Electrical and Electronic Engineering Yonsei University Seoul Republic of Korea; ^2^ Department of Integrated Science and Engineering Yonsei University Incheon Republic of Korea

**Keywords:** contact resistance, energy harvesting, personal thermal management, thermal transport engineering, thermoelectric generator, wearable thermoelectric device

## Abstract

Thermoelectric devices (TEDs) offer a compelling solution by enabling solid‐state, electrically driven thermal regulation directly on the human body. At the same time, they can function as power sources for small electronic devices by utilizing the temperature difference between the body and the surrounding environment. However, the inherently rigid components used in TEDs limit their ability to conform seamlessly to the curved and dynamic surfaces of the human body. This mismatch often results in interfacial gaps and mechanical delamination during motion, significantly degrading thermal and electrical performance. To overcome these limitations, extensive research efforts have been directed toward structural and material design strategies that enhance the mechanical wearability of TEDs for reliable on‐body operation. Here, we review recent advances in wearable TEDs (w‐TEDs), highlighting emerging strategies to reconcile mechanical wearability with high device performance. Structural and material innovations that enable mechanical compliance and conformal skin integration are first reviewed, followed by approaches that improve device performance through electrical contact and optimized thermal transport design. We further examine emerging applications in personal thermal management, energy harvesting, and sensing and discuss the remaining challenges and future opportunities for next‐generation w‐TEDs that simultaneously achieve wearability and high performance.

## Graphical abstract

This review introduces wearable thermoelectric devices as body‐integrated systems rather than isolated energy devices. It explains how their performance depends not only on thermoelectric materials but also on skin contact, mechanical compliance, electrical interfaces, and heat‐flow design. This perspective highlights key design principles for developing practical thermoelectric systems that operate reliably on the human body.

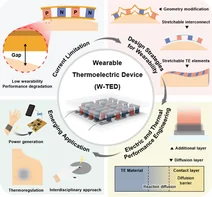

## 1 Introduction

Escalating fossil fuel consumption continues to drive greenhouse gas emissions and accelerate climate change on a global scale [1, 2]. The rapid proliferation of energy‐intensive technologies, including artificial intelligence (AI) and large‐scale data infrastructure, further amplifies cumulative emissions within a fossil‐fuel‐dependent energy system [3, 4]. As global temperatures continue to rise, extreme weather events such as heat and cold waves are becoming more frequent and severe, increasing thermal stress in daily life [5, 6, 7]. Conventional thermal management relies on space‐level conditioning systems, such as air conditioning, which regulate entire environments rather than individuals. However, thermal comfort varies widely depending on physiology, metabolic rate, clothing, and activity [8, 9, 10]. Consequently, ambient conditioning wastes substantial energy while failing to address individual thermal needs [11]. These limitations have motivated the development of personal thermal management (PTM), which regulates temperature locally at the human body [12]. Effective PTM requires compact, lightweight systems capable of precise, rapid, and reversible thermal modulation while remaining compatible with natural body motion [13]. Many existing PTM solutions, including heating garments, fan‐assisted and fluidic‐based cooling devices, remain bulky and typically offer only unidirectional temperature control with limited spatial and temporal resolution [14, 15, 16]. In response to these constraints, wearable thermoelectric devices (w‐TEDs) have emerged as promising candidates for next‐generation PTM systems.

Unlike conventional systems, thermoelectric devices (TEDs) enable solid‐state, electrically controlled heat pumping with no moving parts, offering inherent advantages in reliability and miniaturization [17]. More importantly, their operation is inherently bidirectional: electrical current can drive heat transport for active cooling or heating, while a temperature difference can generate electrical output [18]. This reversible thermoelectric coupling enables a single device architecture to simultaneously support active thermal regulation, energy harvesting, and self‐powered sensing. Consequently, w‐TEDs are increasingly viewed not merely as cooling modules but as multifunctional wearable platforms (Figure 1a) [19, 20, 21, 22]. Despite these advantages, current implementations of w‐TEDs remain largely limited to small, localized modules applied to restricted body regions, such as the neck or wrist, highlighting the need to improve mechanical wearability while maintaining sufficient thermoelectric performance.

**FIGURE 1:**
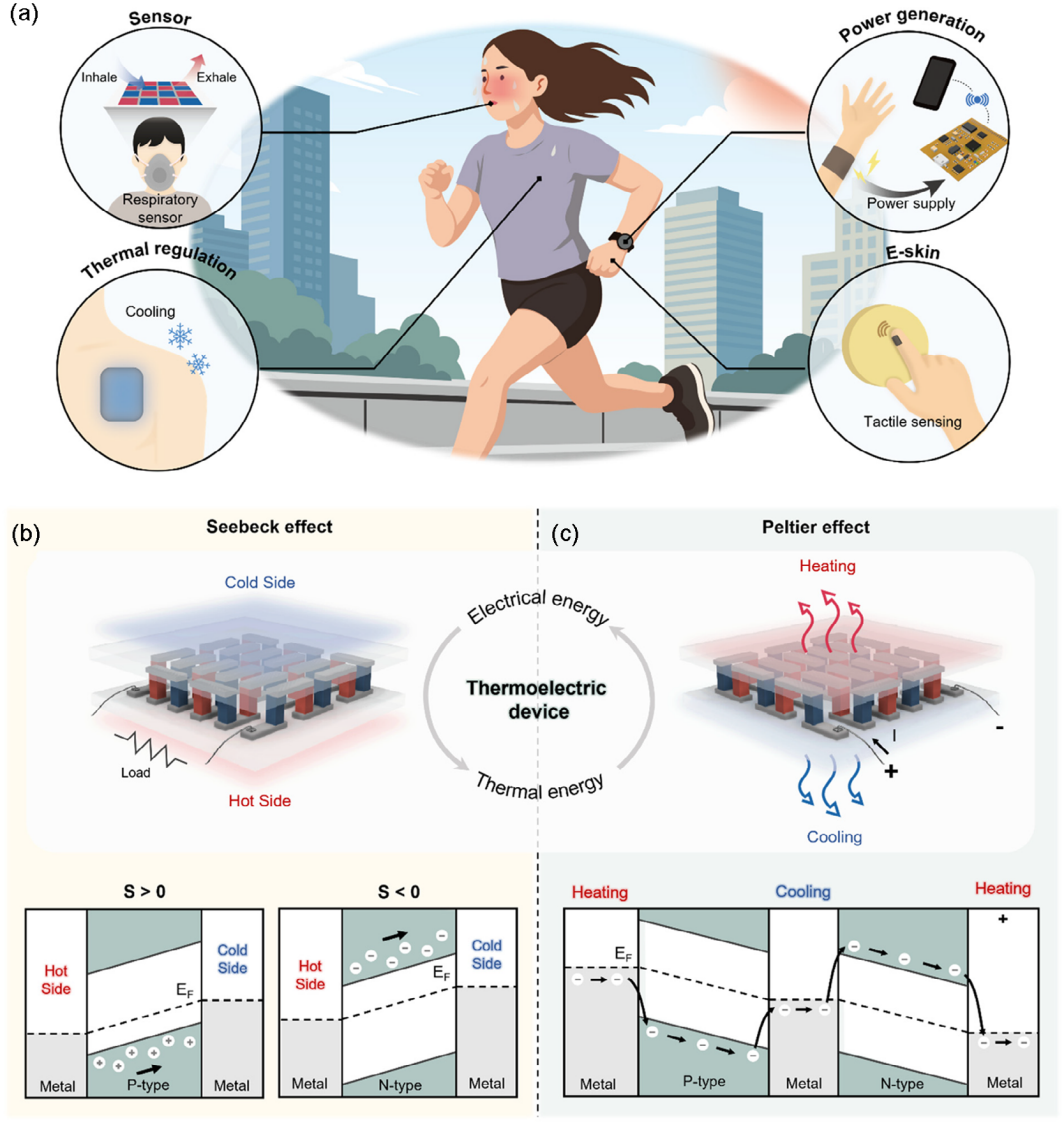
Applications and operating principles of wearable thermoelectric devices (w‐TEDs). (a) Applications of w‐TEDs, including power supply, thermal regulation, physiological sensing, and tactile interfaces. (b) The Seebeck effect for electrical power generation from a temperature difference, and (c) the Peltier effect for active heating and cooling under electrical bias.

To address this need, this review discusses recent advances in w‐TEDs, with particular emphasis on strategies that simultaneously enhance mechanical wearability and device performance. While previous reviews have often focused on thermoelectric material systems, their intrinsic properties, or specific applications, this review systematically examines wearability‐enhancement strategies from a device‐level perspective. These strategies are organized into structural design, stretchable interconnects, and deformable thermoelectric materials. Because increased mechanical compliance can introduce additional electrical and thermal losses, we also discuss complementary strategies for thermal management and the control of electrical and thermal resistances. By integrating these mechanical, electrical, and thermal considerations, this review provides a comprehensive framework for improving wearability while preserving the device performance. Based on this framework, we first summarize the key parameters governing the thermoelectric performance under wearable operating conditions. We then examine the three major approaches for enhancing wearability, followed by strategies to optimize the electrical and thermal performance at the device level. Finally, we highlight emerging applications in PTM, energy harvesting, and self‐powered sensing and outline future perspectives for next‐generation thermoelectric wearable interfaces.

## 2 Thermoelectric Effects

Understanding the operation of w‐TEDs and the factors governing their performance requires examining the fundamental thermoelectric effects. TEDs convert thermal and electrical energy through two complementary phenomena: the Seebeck effect and the Peltier effect. The Seebeck effect enables power generation from a temperature difference, whereas the Peltier effect enables electrically driven heating and cooling. In practical devices, these effects are implemented using p‐type and n‐type thermoelectric legs that are typically connected electrically in series and thermally in parallel, allowing simultaneous control of charge and heat transport.

### 2.1 Seebeck Effect

The Seebeck effect refers to the generation of an electric potential in response to a temperature difference across a thermoelectric material without an externally applied current. In w‐TEDs, this effect enables the direct conversion of the naturally occurring temperature difference between the human body and the surrounding environment into electrical energy. At the charge carrier level, a temperature difference drives thermal diffusion of carriers. Carriers at the hot junction possess higher thermal energy than those at the cold junction. Consequently, these energetic holes in p‐type materials and electrons in n‐type materials diffuse toward the cooler region to reach a lower energy state [23]. This redistribution of carriers creates an internal electric field, which manifests as an open‐circuit voltage (*V*_OC_) (Figure 1b). The resulting voltage is expressed as

(1) $$V_{\mathrm{OC}}=\alpha \text{Δ}T_{\mathrm{leg}}$$

where Δ*T*_leg_ is the temperature difference across the legs, and *α* is the effective device‐level Seebeck coefficient. For a TED composed of p–n thermoelectric pairs connected electrically in series, *α* is given by

(2) $$\alpha =N(S_{p}-S_{n})$$

where *S* denotes the intrinsic Seebeck coefficient of a thermoelectric material, *S*_p_ and *S*_*n*_ are the Seebeck coefficients of the p‐type and n‐type materials, respectively, and *N* is the number of p–n thermoelectric leg pairs in the device. Accordingly, *α* represents the effective device‐level Seebeck coefficient of the electrically series‐connected thermoelectric pairs. In practical wearable systems, however, the temperature difference between the human body and the surrounding environment is distributed across the skin, device interfaces, structural layers within the device, and the external heat‐dissipation pathway owing to their respective thermal resistance. Consequently, the effective temperature difference established across the thermoelectric legs is generally smaller than the overall body–environment temperature difference.

When an external load is connected, the electrical power output of a TED is determined by its *V*_OC_, internal electrical resistance, and load resistance. The output power (*P*) is given by

(3) $$P=\frac{V_{\mathrm{OC}}^{2}\cdot R_{L}}{{\left(R_{\mathrm{TED}}+R_{L}\right)}^{2}}$$

where *R*_L_ is the load resistance, and *R*_TED_ is the internal electrical resistance of the TED. The maximum output power (*P*_max_) occurs under the resistance matching condition (*R*_L_ *= R*_TED_)

(4) $$P_{\max}=\frac{V_{\mathrm{OC}}^{2}}{4R_{\mathrm{TED}}}$$

These relations show that both a high voltage and a low internal resistance are essential for effective power generation.

These device‐level relations describe the determinants of the electrical output. However, the intrinsic origins of voltage generation, charge transport, and parasitic heat leakage are governed by the thermoelectric properties of the constituent materials. These characteristics are commonly described by the dimensionless figure of merit

(5) $$zT=\frac{S^{2}\sigma T}{\kappa }$$

where *S* is the intrinsic Seebeck coefficient of an individual thermoelectric material, *σ* is the electrical conductivity, *κ* is the thermal conductivity, and *T* is absolute temperature. A high *zT* requires a large *S* and high *σ* while maintaining low *κ*. Therefore, *zT* reflects the intrinsic balance among voltage generation, charge transport, and parasitic heat conduction in a thermoelectric material. A related material‐level metric is the power factor (PF)

(6) $$\mathrm{PF}=S^{2}\sigma$$

which evaluates the electrical power‐generation potential of a thermoelectric material. Because it does not account for thermal conductivity, it primarily reflects the electrical performance of the material rather than the full thermoelectric performance.

In wearable devices, the available temperature difference between the human body and the environment is typically small [24, 25]. Under such conditions, the electrical output of the device is inherently limited, and efficient utilization of a small thermal gradient becomes especially important [26, 27]. Therefore, the practical energy‐harvesting performance of w‐TEDs is determined by the interplay between intrinsic material properties and device‐level electrical and thermal characteristics.

### 2.2 Peltier Effect

The Peltier effect describes the absorption or release of heat at the junction between two dissimilar conductors or semiconductors when a current is passed through the junction. In TEDs, this effect enables active heat pumping, such that heat is absorbed at one junction and released at the other. As charge carriers cross the junction, their change in energy state gives rise to localized cooling at one side of the device and localized heating at the opposite side (Figure 1c) [28]. The rate of heat transported by this process is proportional to the applied current. Through the Kelvin relation, it can be expressed as

(7) $$Q_{p}={П}_{\mathrm{eff}}I=\alpha IT$$

where *П*_eff_ is the effective Peltier coefficient of the module, *I* is the applied current, and *T* is the absolute temperature.

In practical TEDs, especially in a wearable cooling configuration, the cold side of the TED is typically placed in contact with the skin, whereas the hot side faces the ambient environment or is coupled to a heat‐dissipation layer. The cold side absorbs heat from the skin and transports it through the TED toward the hot side. The net heat removed from the skin and absorbed at the cold side is denoted by *Q*_C_, whereas the total heat rejected from the hot side to the surrounding environment is denoted by *Q*_H_. Under 1D steady‐state conditions, these heat transfer rates can be expressed as

(8) $$Q_{C}=\alpha IT_{C}-\frac{1}{2}I^{2}R_{\mathrm{TED}}-K\text{Δ}T$$

(9) $$Q_{H}=\alpha IT_{H}+\frac{1}{2}I^{2}R_{\mathrm{TED}}-K\text{Δ}T$$

where *T*_C_ and *T*_H_ are the cold and hot side temperatures, respectively, *K* is its thermal conductance, and Δ*T* = *T*_H_ – *T*_C_. The thermal conductance can be related to the thermal conductivity and device geometry as

(10) $$K=\frac{k_{\mathrm{eff}}A}{L}$$

where *k*_eff_ is the effective thermal conductivity, *A* is the cross‐sectional area for heat transfer, and *L* is the distance between the hot and cold surfaces. In Equations (8) and (9), the first term represents useful Peltier heat transport at the cold and hot sides, respectively. The second term indicates Joule heat generated within the thermoelectric legs, which is assumed to be equally distributed toward the two sides. Accordingly, Joule heating reduces the net cooling capacity at the cold side while increasing the heat rejected at the hot side. The third term represents parasitic heat conduction from the hot side to the cold side. Therefore, *Q*_c_ represents the net cooling capacity after accounting for Joule heating and conductive heat leakage, rather than the Peltier heat alone. In contrast, *Q*_H_ represents the total heat that must be dissipated from the hot side to the ambient environment, including both the heat transported from the cold side and the electrical power (*P*_el_). Therefore,

(11) $$Q_{H}=Q_{C}+P_{\mathrm{el}}$$

where

(12) $$P_{\mathrm{el}}=Q_{H}-Q_{C}=\alpha I\text{Δ}T+I^{2}R_{\mathrm{TED}}$$

The performance of thermoelectric heating and cooling systems is commonly evaluated using the coefficient of performance (COP), defined as the ratio of useful heat transfer to the electrical input power

(13) $$\mathrm{COP}=\frac{Q}{P_{\mathrm{el}}}$$

Accordingly, the cooling and heating COPs are expressed as

(14) $${\mathrm{COP}}_{\text{cooling}}=\frac{Q_{C}}{P_{\mathrm{el}}}=\frac{\alpha IT_{C}-\frac{1}{2}I^{2}R_{\mathrm{TED}}-K\text{Δ}T}{\alpha I\text{Δ}T+I^{2}R_{\mathrm{TED}}}$$

(15) $${\mathrm{COP}}_{\text{heating}}=\frac{Q_{H}}{P_{\mathrm{el}}}=\frac{\alpha IT_{H}+\frac{1}{2}I^{2}R_{\mathrm{TED}}-K\text{Δ}T}{\alpha I\text{Δ}T+I^{2}R_{\mathrm{TED}}}$$

These expressions show that the COP depends on the applied current. Although the Peltier heat‐transport term increases linearly with *I*, Joule heating increases with *I* ^2^. Therefore, increasing the current does not necessarily improve the COP, and an optimum current exists at which the COP is maximized.

By differentiating the cooling COP with respect to the current and applying the optimal current condition, the maximum cooling COP can be expressed as

(16) $${\mathrm{COP}}_{\text{cooling},\max}=\frac{T_{C}}{T_{H}-T_{C}}\frac{\sqrt{1+ZT_{m}}-T_{H}/T_{C}}{\sqrt{1+ZT_{m}}+1}$$

where *Z* = *α* ^2^ */*(*R*_TED_·*K*) is the effective device‐level figure of merit of the TED, and *T*_m_ *=* (*T*_H_ *+ T*_C_)*/2* is the mean operating temperature. The first term corresponds to the Carnot cooling COP, whereas the second term accounts for the reduction caused by the finite thermoelectric performance of the device. As *ZT*_m_ approaches infinity, the second term approaches unity, and COP_max_ approaches Carnot COP. Accordingly, the Carnot cooling COP represents the theoretical upper limit for an ideal reversible system

(17) $${\mathrm{COP}}_{\text{cooling},\text{Carnot}}=\frac{T_{C}}{T_{H}-T_{C}}$$

where *T*_H_ and *T*_C_ are the hot‐side and cold‐side temperatures, respectively.

For heating operation, the COP is related to the cooling COP through the steady‐state energy balance

(18) $${\mathrm{COP}}_{\text{heating}}=\frac{Q_{H}}{P_{\mathrm{el}}}=\frac{Q_{C}+P_{\mathrm{el}}}{P_{\mathrm{el}}}={\mathrm{COP}}_{\text{cooling}}+1$$

Accordingly, under the corresponding optimal operating condition,

(19) $${\mathrm{COP}}_{\text{heating},\max}={\mathrm{COP}}_{\text{cooling},\max}+1$$

In the reversible limit, the maximum heating COP approaches the Carnot heat COP

(20) $${\mathrm{COP}}_{\mathrm{heating},\mathrm{Carnot}}=\frac{T_{H}}{T_{H}-T_{C}}$$

These relations indicate that thermoelectric thermal regulation is highly sensitive to the temperature difference across the device. In wearable systems, where power consumption, battery capacity, and sustained on‐body operation are all critical constraints, a high COP is essential for achieving effective heating or cooling with minimal electrical input. Accordingly, improving the Peltier performance of w‐TEDs requires not only materials with high *zT* but also device structures that minimize parasitic heat loss and interfacial resistance under realistic wearable conditions.

## 3 Strategies for Wearable TEDs

Early w‐TEDs primarily adopted π‐shaped architectures in which rectangular p‐ and n‐type inorganic thermoelectric (ITE) legs with high PF and *zT* values were connected by highly conductive metal electrodes [29, 30]. In these designs, wearability was achieved by encapsulating rigid thermoelectric modules within soft elastomers [31]. However, such approaches were insufficient to accommodate large deformation and ensure conformal contact with the human body under wearable conditions due to the intrinsic rigidity of the thermoelectric device architecture. Recognizing these limitations, subsequent research has sought to improve wearability by fundamentally enhancing the device stretchability through structural and material modifications (Figure 2). These strategies range from geometry modifications that preserve conventional ITE legs and metal electrodes to approaches that replace rigid metal interconnects with intrinsically stretchable conductors. More recent studies further extend this concept by reconfiguring the thermoelectric elements themselves, either through alternative inorganic architectures or by introducing intrinsically stretchable organic thermoelectric (OTE) materials.

**FIGURE 2:**
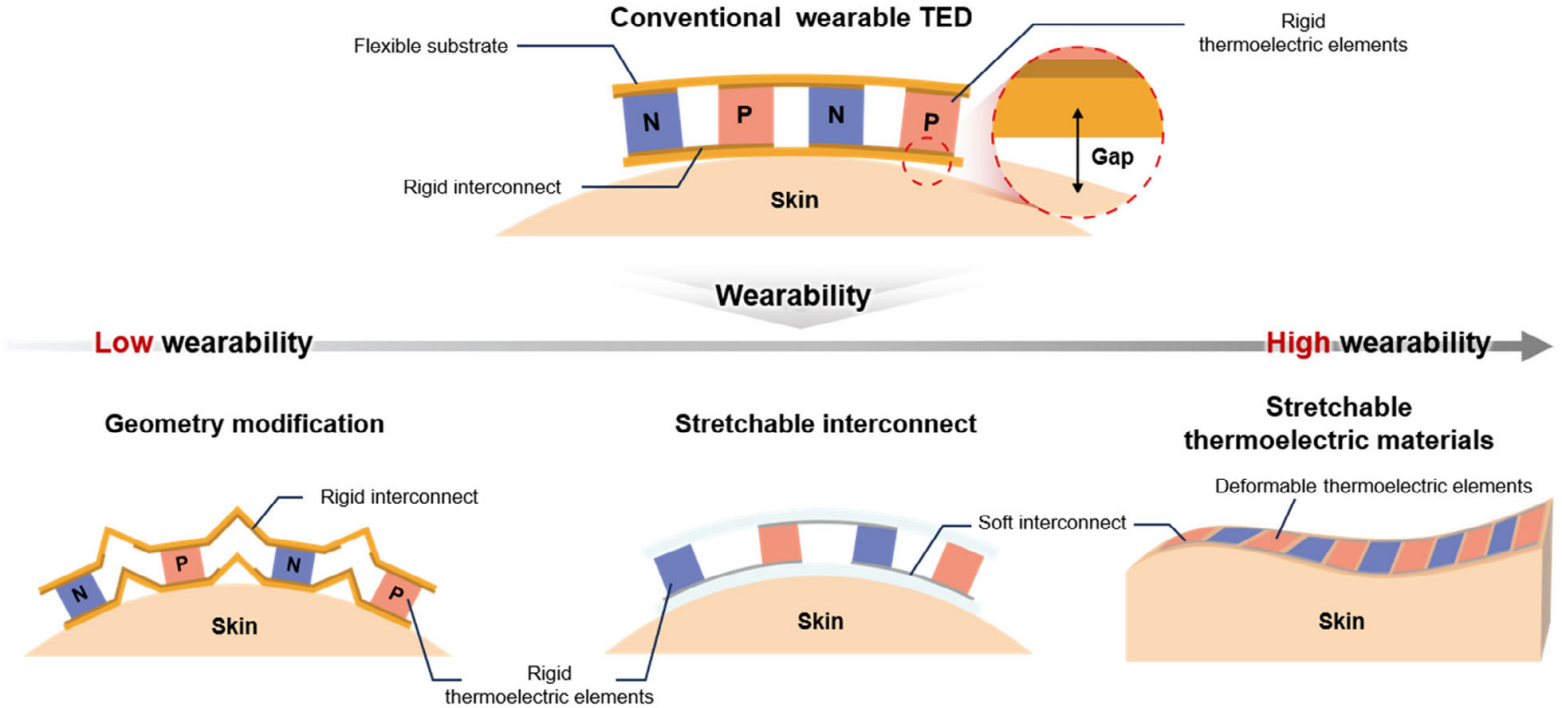
Strategies to enhance the wearability of w‐TEDs. Wearability is progressively improved from poor conformal configurations to highly compliant architectures through geometry modification, stretchable interconnect, and deformable thermoelectric‐element designs that minimize the interfacial gaps with the skin.

### 3.1 Geometry Modification

Many studies employed geometrically engineered interconnects to enhance the stretchability of w‐TEDs. Yang et al. embedded Sb_2_Te_3_ for p‐type and Bi_2_Te_3_ for n‐type cuboids in Ecoflex and electrically interconnected them using serpentine‐shaped electrodes (Figure 3a) [32]. This design achieved biaxial stretchability of over 50%, enabling intimate contact to dynamic curved surfaces such as the elbow (Figure 3b). At Δ*T* = 19.7 K, the device reached a voltage of 117 mV and an output power density of 0.15 mW cm^−2^, demonstrating efficient energy harvesting from waste heat even at complex and highly curved joints (Figure 3c).

**FIGURE 3:**
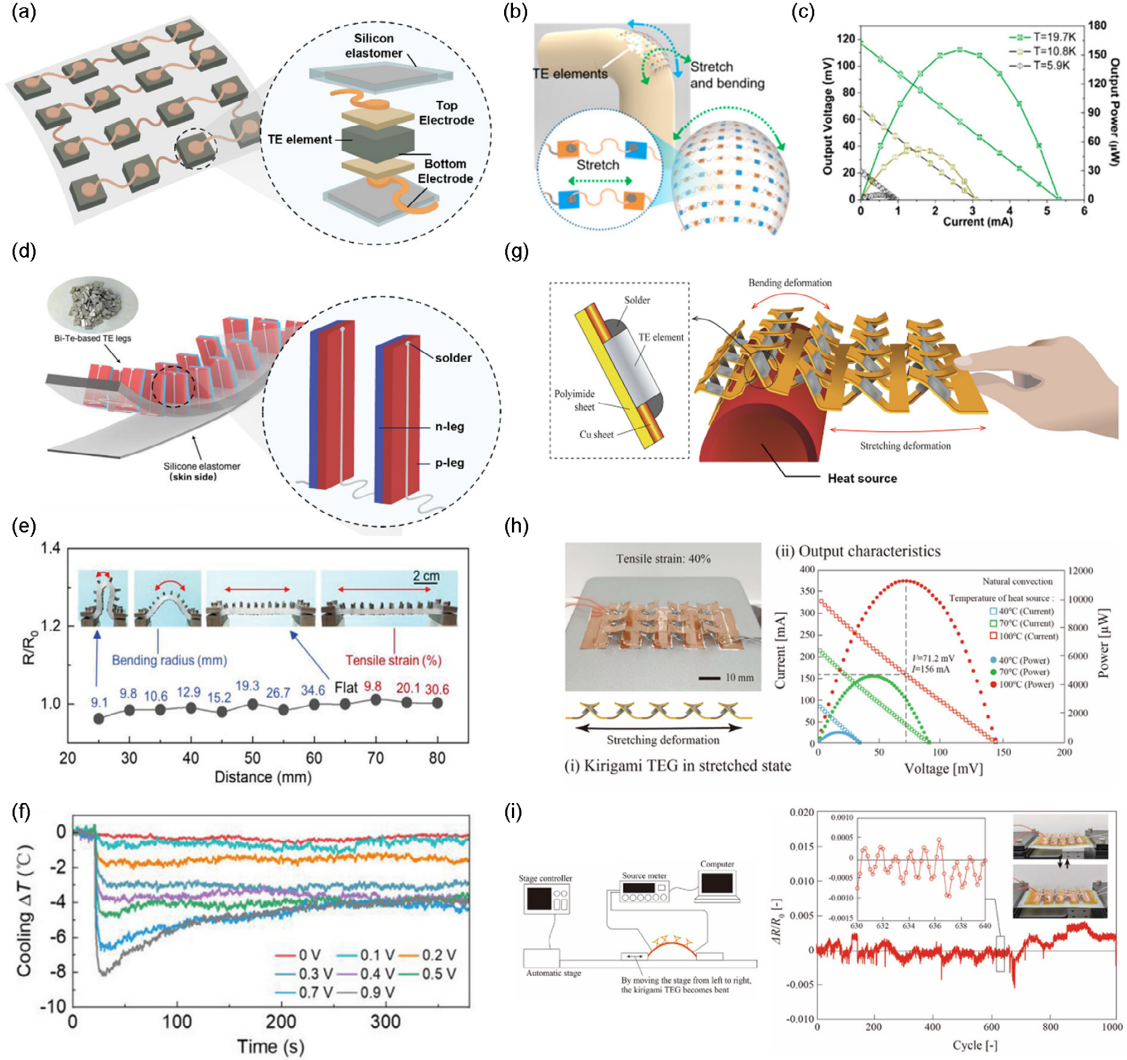
Geometry modification strategies. (a) An illustration of the w‐TED consisting of serpentine electrode integrated with rigid thermoelectric elements. (b, c) Conformal attachment of the w‐TED on a curved surface and the corresponding output voltage and power density at Δ*T* = 19.7 K on the curved surface. Reproduced with permission [32]. Copyright 2020, ACS Publications. (d) Schematic illustration of the fabricated asymmetrically encapsulated w‐TED and (e) photographs demonstrating its stretchability under bending and tensile deformation. (f) Cooling performance of w‐TED under different voltages. Reproduced with permission [33]. Copyright 2024, Wiley‐VCH GmbH. (g) Illustration of the pop‐up kirigami structured w‐TED conformally attached to a cylindrical heat source. (h) Optical image of the stretched device upon 40% tensile strain and the output characteristics according to temperature difference. (i) Resistance variation during cyclic stretching test with an illustration of the experimental setup. Adapted under the terms of the CC‐BY 4.0 License [34]. Copyright 2025, The Authors, published by Springer Nature.

Beyond interconnect‐level modifications, stretchability can be achieved through device‐level structural transformation that fundamentally alters how the entire architecture accommodates deformation. Wu et al. introduced a w‐TED that integrates serpentine wiring with an asymmetrically encapsulated architecture, leaving the top surface exposed while sealing the skin‐contact side (Figure 3d) [33]. This configuration improves bending adaptability by allowing greater structural deformability. With this design, resistance changes remained within 10% during bending at a radius of 9.1 mm. Under a tensile strain of 30.6%, the resistance variation was further reduced to within 5% (Figure 3e). Removing the top encapsulation layer also enhances the cooling efficiency by promoting air convection around the thermoelectric legs. A stable temperature drop of 5 K was maintained at an applied voltage of 0.5 V (Figure 3f), while a power density of 4 μW cm^−2^ was generated at Δ*T* = 27 K under a 2 m s^−1^ airflow. While asymmetric encapsulation improves mechanical compliance by partially releasing structural constraints, the overall deformability of the device remains limited by its continuous, substrate‐supported architecture. To overcome this limitation, Terashima et al. introduced a strategy to improve wearability by implementing a pop‐up kirigami structure in a flexible TED formed by laser cutting (Figure 3g) [34]. The device was conformally attached to a cylindrical heat source at 100 °C, exhibiting a *V*_OC_ of 144 mV and a *P*_max_ of 12.4 mW. Even under 40% tensile strain, the device maintained stable contact with the heat source and preserved similar performance, generating comparable *V*_OC_ (143 mV) and output power (11.1 mW), as shown in Figure 3h. In addition, the resistance variation was less than 1% even at tensile strains up to 70%, and the internal electrical resistance was further examined under repeated mechanical deformation. Even after 1000 deformation cycles, no noticeable performance degradation or resistance drift was observed. The resistance change remained approximately 0.2%, with only ~1% instantaneous variation during deformation (Figure 3i). These results indicate that no structural damage occurred in the thermoelectric elements, solder joints, or substrate, demonstrating high mechanical stability and robustness of the pop‐up kirigami TED architecture.

ITE materials generally provide high thermoelectric performance but are too rigid to directly accommodate body deformation. Geometry modification is used to retain these high‐performance materials while allowing the device to adapt to the body motion. However, local stress concentration near the rigid thermoelectric elements and their interface, loss of active area, and nonuniform thermal contact remain unsolved. Accordingly, tailoring the device architecture to the deformation characteristics of the target body site would be more effective than pursuing maximum stretchability alone.

### 3.2 Stretchable Interconnect

Replacing rigid metal wiring with soft interconnects sacrifices the structural rigidity that previously stabilized the thermoelectric leg array. As a result, mechanical deformation can lead to the collapse or misalignment of the thermoelectric leg array, causing structural instability. Therefore, a key challenge in replacing rigid metal interconnects with soft conductors is to simultaneously maintain high device performance while ensuring the structural stability of the thermoelectric leg array.

Liquid metal (LM) is widely used as a stretchable interconnect in wearable devices due to its exceptional electrical conductivity and mechanical deformability. Zadan et al. reported a device structure employing a LM‐embedded elastomer (LMEE), which functions as both the electrical interconnect and the heat‐dissipation medium (Figure 4a) [35]. In this system, electrical conductivity is activated by applying localized pressure to specific areas of the LMEE composite, resulting in a conductivity of 1.37 × 10^5^ S m^−1^, establishing it as a viable high‐conductivity alternative to rigid metal electrodes. At a temperature difference of Δ*T* = 60 K, the device achieved a *V*_OC_ of 278.6 mV (Figure 4b). Under the same temperature difference, the output voltage was measured as a function of the load resistance, as shown in Figure 4c. The *P*_max_ was obtained at a load resistance of 11.5 Ω, suggesting an internal device resistance of approximately 11.5 Ω based on impedance matching. In this design, pure polydimethylsiloxane (PDMS) is utilized as a filler material between the thermoelectric legs to stabilize the structure. However, PDMS has a thermal conductivity of 0.16 W m^−1^ K^−1^ at 25 °C, which is approximately 6–8 times higher than that of air (0.024 W m^−1^ K^−1^ at room temperature) [38, 39]. This mismatch creates parasitic thermal pathways that shunt heat through the filler rather than the thermoelectric legs, degrading the effective temperature difference and overall device efficiency. To mitigate this issue, Ramesh et al. introduced an aerogel‐silicone composite filler composed of aerogel particles dispersed in PDMS (Figure 4d) [36]. Aerogel particles with sizes ranging from 100 to 700 μm were incorporated at a volume fraction below 50%, resulting in a linear reduction in thermal conductivity while maintaining a low Young's modulus (Figure 4e). Consequently, the optimized composite provided both thermal insulation and mechanical reliability. The device exhibited negligible changes in electrical resistance even after 100 bending cycles at a bending radius of 6 mm. Due to the significantly reduced thermal conductivity (0.08 W m^−1^ K^−1^) of the filler, the device achieved a peak *V*_OC_ of 70 mV at Δ*T* = 11.5 K, as illustrated in Figure 4f.

**FIGURE 4:**
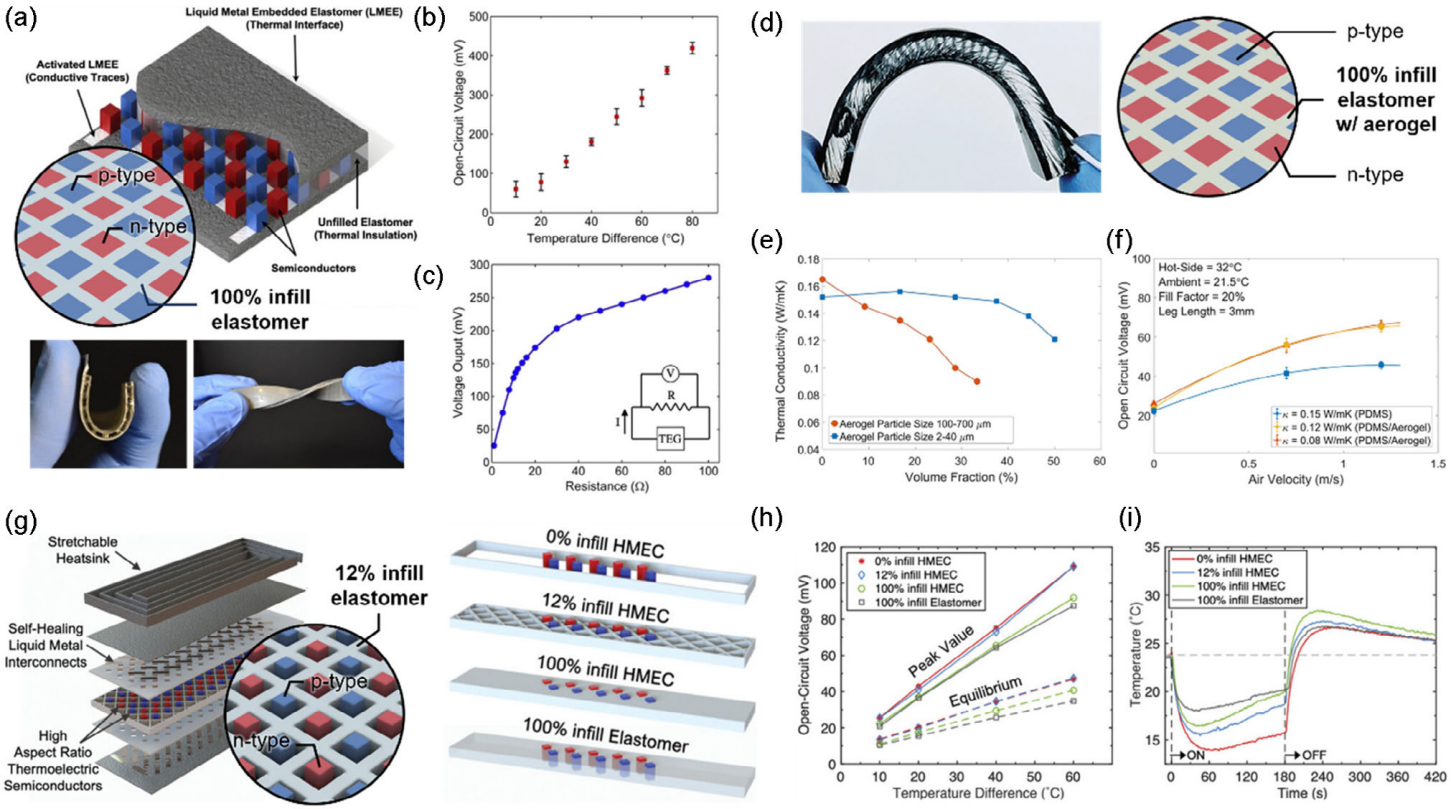
Stretchable interconnects and filler‐material engineering. (a) Schematic of the liquid metal‐embedded elastomer (LMEE) with 100% pure polydimethylsiloxane (PDMS) filling material and flexibility under various deformations. (b) Open‐circuit voltage (*V*_OC_) at different temperature differences. (c) Output voltage as a function of load resistance. Reproduced with permission [35]. Copyright 2020, ACS Publications. (d) Illustration of the device incorporating an aerogel‐PDMS composite filler. (e) Thermal conductivity of the composite as a function of aerogel particle size and volume fraction. (f) *V*_OC_ as a function of air velocity for fillers with different thermal conductivities. Reproduced under the terms of the CC‐BY 4.0 License [36]. Copyright 2021, The Authors, published by Springer Nature. (g) Device illustration incorporating a hollow microsphere elastomer composite (HMEC) stretchable thermal insulator. (h) *V*_OC_ as a function of Δ*T* for different infill ratios. (i) Temperature response under on–off operation at different infill ratios. Reproduced with permission [37]. Copyright 2024, The Authors, published by Wiley‐VCH GmbH.

Despite the reduced thermal conductivity, the aerogel–PDMS composite still exhibits poorer thermal insulation than air. Consequently, the most effective approach to concentrating heat flux through the thermoelectric legs is to enable direct contact between air and the thermoelectric legs. Han et al. introduced a stretchable thermal insulator based on a hollow microsphere elastomer composite (HMEC) with a 12% infill ratio, which provides sufficient structural support to preserve the geometry while enabling the formation of air pockets (Figure 4g) [37]. This architecture effectively mitigated parasitic heat transfer by incorporating air pockets, resulting in a 27% increase in *V*_OC_ compared to that of a fully infilled elastomer (Figure 4h). In addition, this design delivered effective cooling and reduced thermal backflow after power‐off (Figure 4i). By suppressing residual heat transfer to the inactive side, this architecture mitigates rapid temperature rebound and enhances sustained temperature modulation.

Stretchable interconnects are advantageous for devices exposed to repeated tensile deformation, where rigid metal wiring fails. However, the mechanical mismatch between soft interconnects and rigid thermoelectric legs can generate concentrated stress at their interface, leading to debonding or degradation of the electrical contact during deformation. An elastomer filler is often introduced to secure the leg array and distribute mechanical stress. Because conventional fillers have higher thermal conductivity than air, they create parasitic heat‐transfer pathways and reduce the effective temperature difference across the thermoelectric legs. The HMEC‐based architecture mitigates this problem by introducing air pockets while retaining sufficient structural support for the leg array. Nevertheless, because the filler remains between the thermoelectric legs, heat can still bypass the legs through the filler, resulting in lower performance than that of a fully open architecture. Therefore, future research should consider interface designs that maintain robust electrical and mechanical bonding between soft interconnects and rigid thermoelectric legs throughout deformation, thereby reducing reliance on thermally parasitic fillers.

### 3.3 Deformable ITE Materials

The approaches described above primarily modify the surrounding structures rather than the inherently rigid thermoelectric legs themselves. To further enhance wearability, many studies have therefore focused on converting bulk ITE materials into alternative configurations (Table 1). Among these strategies, thin‐film structures represent the most widely explored approach. Tan et al. fabricated a w‐TED using sputtered thin films of Bi_0.__5_Sb_1.__5_Te_3_ and Bi_2_Te_2.__7_Se_0.__3_ for the p‐type and n‐type elements, respectively (Figure 5a) [47]. To enhance electrical contact between the Cu electrodes and the thermoelectric thin films and minimize interfacial fracture, Ti interfacial layers were introduced. The p‐type and n‐type elements were then integrated through flip‐chip bonding, enabling high‐density interconnection of 162 pairs of thermoelectric elements. As a result, the device exhibited a *V*_OC_ of 306.5 mV and an output power of 225.4 μW at Δ*T* = 5 K (Figure 5b). Furthermore, the device showed only ~3% resistance change after more than 100 bending cycles at a bending radius of 10 mm (Figure 5c), demonstrating its reliability under repeated mechanical deformation. Another fabrication strategy based on screen printing was reported by Zhang et al. [48]. After printing Ag and Cu patterns, the films were immersed in a solution containing Se powder and Na_2_S 9H_2_O, where in situ selenization converted the metal films into n‐type Ag_2_Se and p‐type Cu_2_Se (Figure 5d). The 42 pairs of thermoelectric thin films were interconnected using Ag paste to improve mechanical deformability. The devices generated a *V*_OC_ of approximately 110 mV and a *P*_max_ of 14.75 μW at a Δ*T* = 60 K (Figure 5e). The mechanical stability of the device was further evaluated under various bending radii and cycles. At a bending radius of 6 cm, the resistance change remained below 10% even after 500 bending cycles. When it was reduced to 4 and 2 cm, the resistance increased by approximately 15% and 24%, respectively. In addition, *V*_OC_ remained nearly unchanged during bending, and the device retained approximately 81% of its initial power density after 1500 cycles (Figure 5f).

**TABLE 1:** Strategies for transforming bulk ITE materials into wearable architectures.

Material format	p‐type	n‐type	Fabrication method	*V*_OC_, mV	Power	Δ*T*, K	Pairs	References
Nanowires	Bi_0.5_Sb_1.5_Te_3_	Bi_2_Te_3_	Inkjet printing	46	127 nW	32.5	5	[40]
Ink	Bi_0.4_Sb_1.6_Te_3_	Bi_2_Te2.7Se_0.3_	Brush printing	22.5	3 μW	20.1	5.5	[41]
Ink	PEDOT:PSS	Ag_2_Se	Screen printing	71.1	1.72 μW	30	13	[42]
Ink	Bi_0.4_Sb_1.6_Te_3_ + 5 wt% Te	Bi_2_Te_3_ + 7.5 wt% Te	Screen printing	13.8	2.8 mW	20	8	[43]
Paste	(0.25 Bi, 0.75 Sb)_2_(0.95Te,0.05Se)_3_	(0.98 Bi, 0.02 Sb)_2_(0.9Te,0.1Se)_3_	Dispenser printing	23.9	3.107 nW	22.5	12	[44]
Ink	—	Bi_2_Te_2.7_Se_0.3_	Aerosol‐jet printing	25	141 nW	50	—	[45]
Paper composite	Bi_0.5_Sb_1.5_Te_3_	Bi_2_Te_2.7_Se_0.3_	Vacuum‐assisted filtration	38.55	95.17 nW	24	2	[46]

**FIGURE 5:**
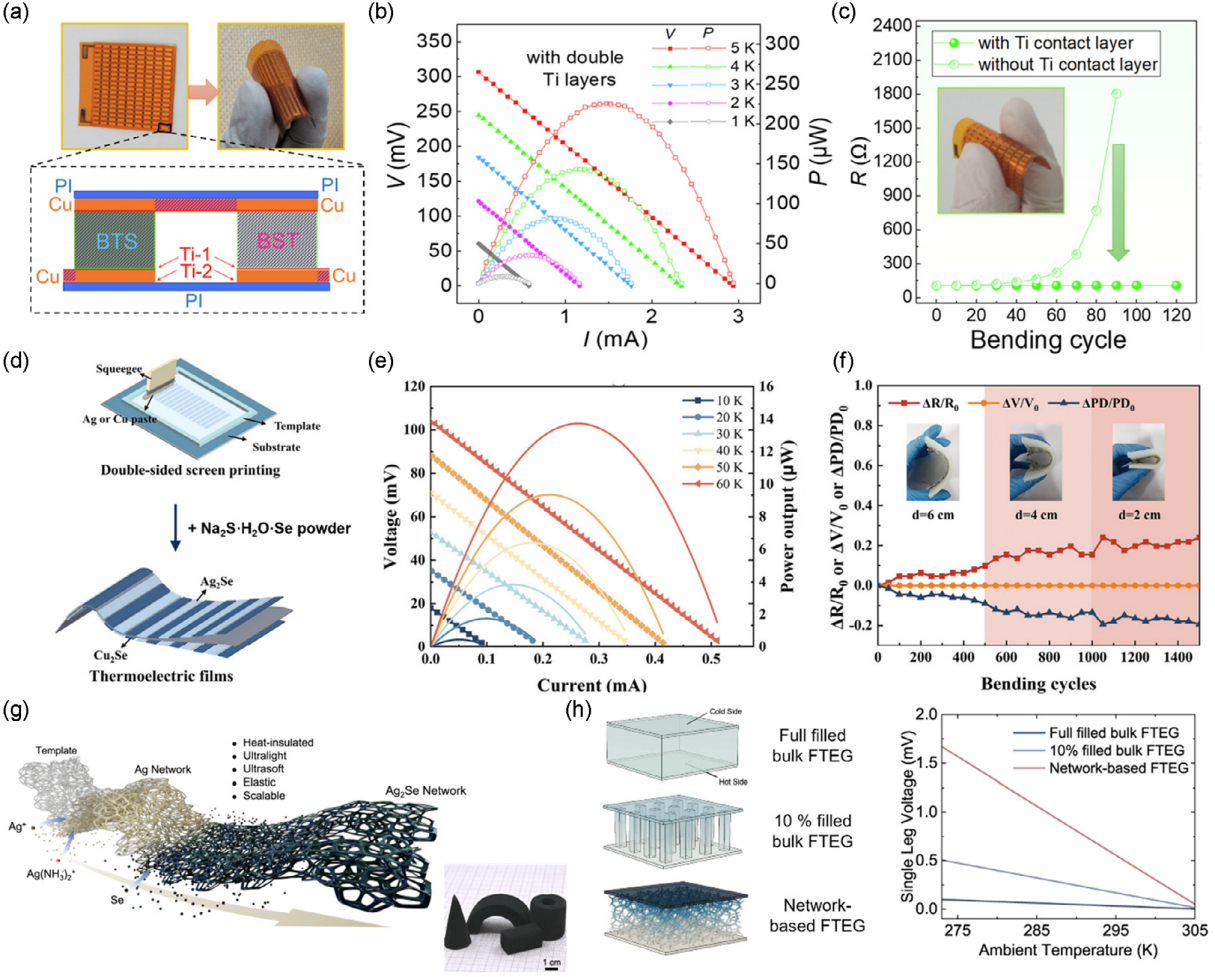
Structural engineering of stretchable ITE materials. (a) Optical image of the fabricated devices using doped thermoelectric materials and the illustration of the thin film‐based device structure. (b) *V*_OC_ and output power of 162‐pair array. (c) Electrical resistance of the devices with and without double Ti layers as a function of bending cycles. Reproduced with permission [47]. Copyright 2025, The Authors, published by Springer Nature. (d) Schematic illustration of the process for in situ synthesis of a double‐sided thermoelectric film on polyimide (PI) substrate. (e) Output performance of the device with 42 pairs under different temperature differences. (f) Relative electrical resistance, voltage, and power under different bending radius and cycles. Adapted with permission [48]. Copyright 2024, Wiley‐VCH GmbH. (g) Schematic of a 3D elastic Ag_2_Se network architecture designed to generate out‐of‐plane temperature differences. (h) Various FTEG architectures and single leg voltage at different ambient temperatures. Reproduced with permission [49]. Copyright 2023, The Authors, published by Springer Nature.

In thin‐film architecture, heat transport often occurs along the in‐plane direction of the films. However, heat flux from the skin to the ambient environment primarily occurs through the device thickness [50]. Thus, thin‐film configuration reduces the effective temperature difference across the thermoelectric elements, thereby limiting thermoelectric conversion efficiency compared with bulk architectures [51]. To overcome this limitation, Liu et al. developed a 3D elastic network by synthesizing Ag_2_Se directly onto a melamine foam template, as illustrated in Figure 5g [49]. This microstructured network redistributes heat‐flow pathways to promote uniform flux along the temperature difference while suppressing parasitic heat loss through nonproductive routes, thereby maintaining a high temperature difference across the device. Compared to conventional full‐filled or 10% infilled bulk flexible thermoelectric generators (FTEGs), the network‐based architecture maintained a superior temperature difference of ~6.5 K, resulting in the maximum single leg voltage among them (Figure 5h). When integrated into clothing, the device generated approximately 1 mW of power while walking in an indoor environment at 290 K, demonstrating its scalability and practical potential as a wearable energy harvester.

Deformable ITE materials generally retain higher thermoelectric performance than OTE materials because their crystalline structures enable more efficient charge transport. Therefore, they are particularly suitable when a high electrical output must be maintained together with bending compliance and moderate deformability. Thin‐film architectures are advantageous for integrated devices, but their predominantly in‐plane thermal pathways are poorly aligned with the out‐of‐plane heat flow from the skin to the ambient environment. 3D networks improve this thermal alignment and preserve a larger temperature difference, although their lower active material fraction and electrical uniformity can limit output. Therefore, future deformable ITE designs should combine the high electrical quality of dense films with vertically aligned or 3D heat flow pathways.

### 3.4 OTE Materials

Despite extensive efforts to engineer various architectures of ITE materials, intrinsic rigidity still limits stretchability. As a result, OTE materials, such as polyaniline (PANI), carbon nanotubes (CNTs), and poly(3,4‐ethylenedioxythiophene):poly(styrene sulfonate) (PEDOT:PSS), have recently emerged as promising alternatives (Table 2). Although OTE materials generally exhibit *S* and PF lower than those of ITE materials, their intrinsic mechanical flexibility and stretchability make them particularly suitable for w‐TEDs. Sun et al. developed a 1D CNT‐based thermoelectric fiber designed to exploit cross‐plane thermal differences in wearable conditions to address these performance limitations of OTE materials [75]. The carbon nanotube fiber (CNTF) was dipped into PEDOT:PSS solution to fabricate p‐type CNTFs, and oleamine doping was used to fabricate n‐type CNTFs (Figure 6a). Regardless of the thermoelectric unit length, the output voltage increased with the increasing temperature difference (Figure 6b). In addition, the shorter thermoelectric units exhibited lower internal resistance, resulting in an increase in output power from 4.4 to 4.64 μW (Figure 6c). Similarly, Lan et al. fabricated p‐type thermoelectric fabrics with an electrical conductivity of 18.8 S cm^−1^ by dipping cotton fabric into a PEDOT:PSS solution [78]. The p‐type CNT fabric was treated with polyethylenimine (PEI) to obtain the n‐type fabric, exhibiting an electrical conductivity of 871 S cm^−1^. The 8‐pair device generated an output voltage of 45.2 mV and a power of 375 μW upon stretching. These results highlight the potential of textile‐based TEDs for robust performance in dynamic wearable environments.

**TABLE 2:** Comparison of thermoelectric properties of OTE and ITE materials.

Materials	Type	*S*, μV K^−1^	*σ*, S cm^−1^	*PF*, μW m^−1^ K^−2^	References
**OTE materials**					
PEDOT:PSS	P	15–18	0.1–1	~0.013–0.08	[52, 53]
PEDOT:PSS/SWCNT	P	20	~1062.8	89	[54]
PEDOT:PSS/CNT	P	44	~28	~5.8	[55]
PEDOT:PSS/DMSO/Na_2_SO_3_	P	~35	>800	105.2	[56]
Undoped PANI (emeraldine base)	—	—	<10^−9^	<1	[57, 58]
Doped PANI (emeraldine salt)	—	—	≥10	—	[59]
CAS‐doped PANI(at 323 K)	P	17.8	14.1	0.4	[60]
PANI/SWCNT	N	~−48	769	176	[61]
PANI/CNTs	N	−42.7	2238	407	[62]
CNT	P	64.5	6440	2619	[63]
CNT/PEI	N	−29.7	11 841	1051	[63]
SDBS‐doped SWCNT	P	~35–64	~1965	145–308	[64]
CTAB‐doped SWCNT	N	~ −38 to −42	~1351	127–258	[64]
PBFDO	N	−21	~2000	~90	[65]
CNT/P3HT on silk fibroin	P	~41.8	~1170	~204	[66]
**ITE materials**					
** Bulk**					
Bi_2_Te_3_	P/N	P: 150–250 N: −50 to −200	~2000	500–4500	[67, 68, 69]
** Thin‐film**					
Bi_2_Te_3_	N	~146	691	1473	[70]
Bi_2_Te_3_	P/N	P: ~184.2 N: ~−198.9	—	P: 1250 N: 1490	[71]
Bi_2_Te_3_	N	130.38	512	871	[72]
Sb_2_Te_3_	P	133.05	1049	1858	[72]
Ag‐doped SnSe	P	207–217	278.8	1197	[73]
Al‐doped Cu_2_Se	P	163.2	—	1075	[74]

Abbreviations: CTAB, cetyltrimethylammonium bromide; P3HT, poly(3‐hexylthiophene); PBFDO, poly(benzodifurandione); SDBS, sodium dodecylbenzene sulfonate.

**FIGURE 6:**
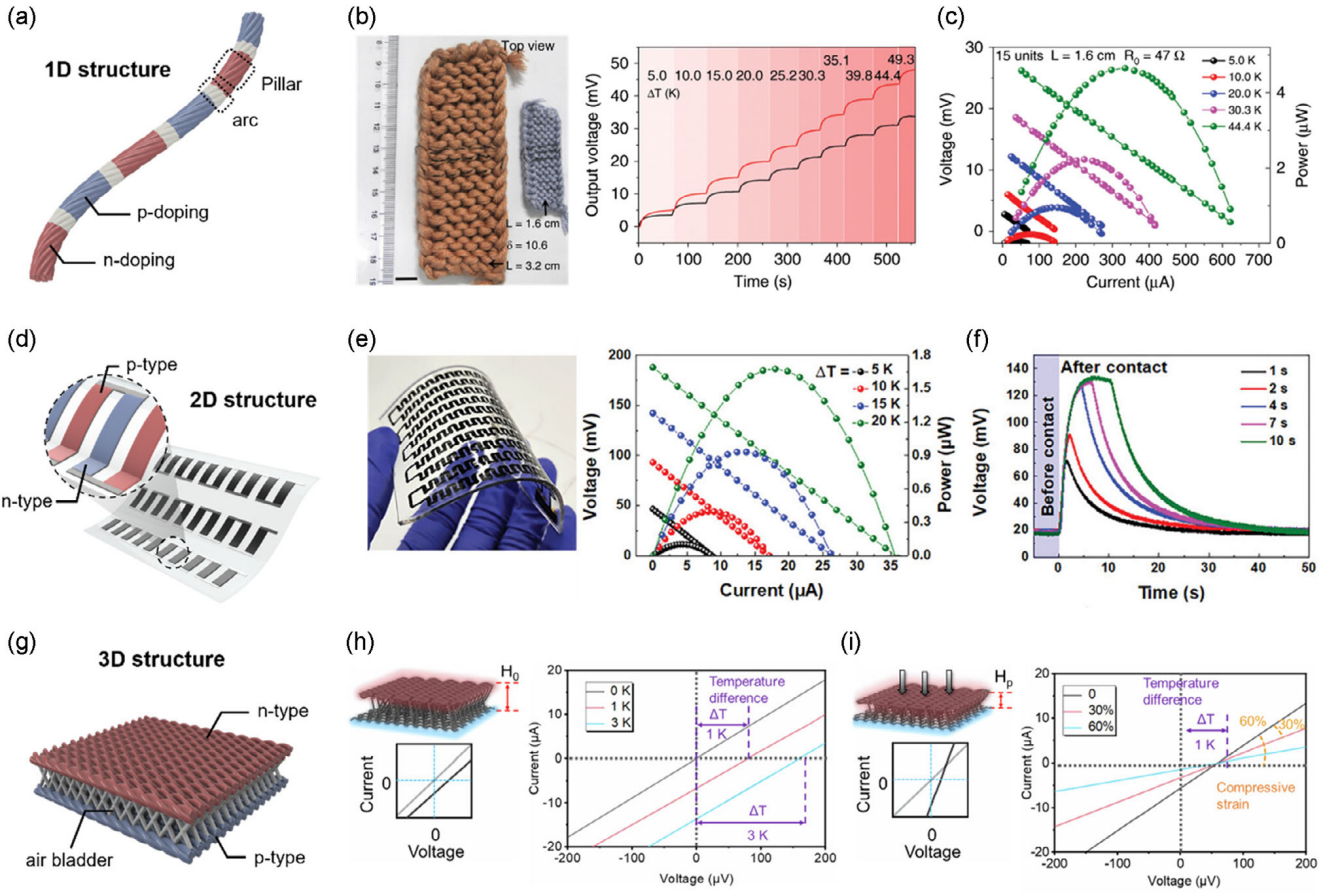
Multidimensional OTE architectures. (a) Schematic illustration of 1D OTE fibers with PEDOT:PSS and oleamine doping for p‐ and n‐type thermoelectric legs, respectively. (b) TED fabricated with different dimensions while maintaining a constant number of thermoelectric legs, and their corresponding output voltages. (c) Output voltage and power as a function of current under various temperature differences for reduced‐dimension devices. Reproduced under the terms of the CC‐BY 4.0 License [75]. Copyright 2020, The Authors, published by Springer Nature. (d) Pre‐doped thermoelectric inks printed onto a shaped PDMS substrate through direct ink writing. (e) Image of a TED composed of 140 p–n couples and its corresponding output performance. (f) Output voltage for various hand contact durations when the TED is attached to the human palm. Reproduced under the terms of the CC‐BY 4.0 License [76]. Copyright 2023, The Authors, published by Wiley‐VCH GmbH. (g) Design concept of a 3D thermoelectric spacer fabric (TESF). (h, i) *I*–*V* curves of TESF under different applied ΔT values and varying tensile strains at a constant ΔT of 1 K. Reproduced with permission [77]. Copyright 2025, The Authors, published by Springer Nature.

However, a 1D configuration may limit the achievable performance because the thermoelectric elements are arranged along the in‐plane direction, which is perpendicular to the heat flow and therefore restricts the effective temperature difference across the device. Hence, Hwang et al. introduced w‐TEDs fabricated by printing thermoelectric inks as planar patterns onto a shaped PDMS insulator (Figure 6d) [76]. p‐ and n‐type inks consisted of single‐walled carbon nanotubes (SWCNTs) doped with poly(acrylic acid) (PAA) and PEI, respectively. This structure features vertically aligned thermoelectric materials on a planar surface, with better alignment to the out‐of‐plane temperature gradient than the 1D configuration. The device with 140 p–n pairs generated a maximum output voltage of 188.08 mV and a power output of 1.68 μW at Δ*T* = 20 K, highlighting the potential for high power density through large‐area scalability (Figure 6e). When the array was attached to a cup of cold water (~4.5 °C) and placed in contact with a human palm, it generated a maximum voltage of 134.11 mV for 7 s (Figure 6f). To improve the thermoelectric performance, 3D configuration has also been explored. It offers significant advantages by eliminating the insulation layer and enabling the decoupling of pressure‐ and temperature‐induced signals. To realize these advantages, He et al. introduced a 3D supporting‐yarn framework to construct a structural framework, and the p‐type fabric was prepared by treatment with PEDOT:PSS, and the constantan wire was used for the n‐type [77]. This architecture results in an exceptionally low thermal conductivity (*κ* < 0.05 W m^−1^ K^−1^), which effectively preserves the out‐of‐plane Δ*T* (Figure 6g). The 3D spacer provided distinct structural paths for thermal and mechanical stimuli. The static air pockets within the 3D structure act as low‐thermal‐conductivity regions that delay rapid thermal equilibration between the hot and cold sides, thereby preserving an effective out‐of‐plane Δ*T* for Seebeck voltage generation. Therefore, changes in Δ*T* are directly reflected in the voltage and appear as a shift in the *I*–*V* curve. In contrast, when pressure is applied, the spacer yarns deform and increase their effective contact area with the upper and lower fabric layers. This pressure‐induced resistance change is manifested as a change in the slope of the *I*–*V* curve. The distinction between the two signal pathways is clearly shown in Figure 6h,i. A temperature difference shifts the output curve without substantially changing the *I*–*V* slope, whereas applied pressure changes the slope through a piezoresistive reduction in the electrical resistance. Finally, the authors demonstrated the high potential of 3D‐architected thermoelectric fabrics for multifunctional wearable electronics by incorporating them into masks and gloves. Zhu et al. proposed a scalable way to fabricate porous p‐type thermoelectric fabrics by coating PEDOT:PSS and SWCNTs onto a hot‐air‐bonded nonwoven scaffold composed of a core‐sheath poly(ethylene terephthalate) (PET), in which the core and sheath have different melting temperatures [79]. The resulting conductive fibrous scaffold served as a 3D porous thermoelectric fabric, allowing dual‐mode sensing in a manner similar to the 3D thermoelectric fabric discussed above. The pressure response was mainly used for human–machine interface (HMI)–related applications, such as pulse monitoring, human motion detection, sign language recognition, and robotic‐arm‐based object recognition, whereas the thermal response was demonstrated by distinguishing cold and hot objects. Liu et al. further extended this 3D‐architected decoupling strategy to underwater environments by developing a waterproof p‐type thermoelectric polyurethane foam (TEPUF) composed of SWCNT, PEDOT:PSS, and waterborne polyurethane (WPU) [80]. Based on this signal‐decoupling capability, the TEPUF was used as an underwater wearable system to monitor three diver‐related physiological and motion signals: respiration, body temperature, and swimming posture/motion. Respiration and swimming motion are detected from the piezoresistive response, where foam deformation increases the pore‐wall contact, thereby changing the electrical resistance. In contrast, the body temperature is monitored from the Seebeck voltage generated by the temperature difference between the human body and the surrounding water.

Overall, these studies demonstrate the versatility of 3D porous or architected structures as platforms for multimodal thermoelectric sensing. The 3D spacer fabric establishes the basic temperature‐pressure decoupling mechanism, the hot‐air‐bonded fibrous scaffold provides a scalable route for porous thermoelectric fabric fabrication, and the waterproof thermoelectric foam extends the same concept to underwater sensing environments. Beyond structural design, advanced PEDOT:PSS modification strategies, including dedoping and π–π overlap engineering [81], can enhance the Seebeck voltage response of the temperature channel, thereby further improving the performance of decoupled multimodal thermoelectric sensors.

OTE materials are particularly suitable for textile‐integrated, highly deformable, and multifunctional wearable systems because of their intrinsic softness, lightweight nature, breathability, and solution processability. However, their practical use remains limited by relatively low electrical conductivity and PF, unstable doping, and inefficient utilization of the out‐of‐plane temperature gradient, which together result in a lower output power than that of most ITE‐based devices. Nevertheless, OTE materials may become a particularly promising material platform for w‐TEDs because their excellent processability and compatibility with textiles and porous structures offer considerable potential for integrated multifunctional devices. Therefore, future research should first focus on improving the overall thermoelectric performance and environmental stability of OTE materials. Particularly, attention should be directed toward high‐performance n‐type OTE materials, which remain considerably less developed than their p‐type counterparts, as well as 3D device architectures that improve thermal transport and enable reliable multimodal signal decoupling.

## 4 Performance Enhancement Strategies

Recent w‐TEDs have adopted new materials and device architectures to enhance wearability, but these strategies often reduce the power density and thermoregulation performance compared with conventional rigid TEDs [82, 83, 84]. For example, replacing rigid metal heat sinks with soft heat sinks may weaken the heat dissipation. Soft interconnects and stretchable electrical joints may increase the electrode and interfacial contact resistance. In addition, soft substrates, fillers, and encapsulation layers can introduce parasitic heat‐flow pathways, reducing the effective temperature difference across the thermoelectric legs. Thus, compensatory design strategies are essential to sustain performance, particularly by minimizing thermal and electrical resistances to preserve the temperature difference under continuous operation [85, 86].

When evaluating thermal resistance, it is imperative to consider the combined effects of interfacial, internal, and external thermal resistance rather than focusing solely on a single component. The overall configuration of the w‐TED and its corresponding thermal circuit is illustrated in Figure 7a. In this model, *R* ^th^_sink_ represents the thermal resistance on the ambient side, including the thermal resistance of the heat sink (*R* ^th^_sink,mat_) and the heat‐transfer resistance between the heat sink surface and the surrounding environment (*R* ^th^_sink‐amb_). *R* ^th^_TED_, *R* ^th^_skin_, and *R* ^th^_c,skin_ correspond to the thermal resistance of the TED, the skin and underlying tissue, and the skin–device interface, respectively. According to the heat‐transfer equation, the total heat flow from the body tissue to the ambient air can be expressed as

**FIGURE 7:**
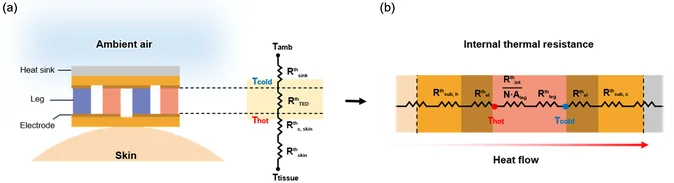
Thermal resistance network of a w‐TED. (a) Schematic illustration of the overall w‐TED configuration and its corresponding thermal resistance circuit. (b) Detailed internal thermal resistance model for the w‐TED, decomposing the total resistance into contributions from the hot‐side substrate, electrodes, interfaces, thermoelectric legs, and cold‐side substrate.

(21) $$Q_{\text{total}}=\frac{\text{Δ}T_{\text{total}}}{{R^{\mathrm{th}}}_{\text{total}}}$$

where Δ*T*_total_ indicates the temperature difference between the effective body‐tissue temperature and the ambient air, and *R* ^th^_total_ = *R* ^th^_skin_ + *R* ^th^_c,skin_ + *R* ^th^_TED_ + *R* ^th^_sink_. Similarly, the heat flow through the w‐TED can be written as

(22) $$Q_{\mathrm{TED}}=\frac{\text{Δ}T_{\mathrm{TED}}}{{R^{\mathrm{th}}}_{\mathrm{TED}}}$$

where Δ*T*_TED_ denotes the temperature difference between the hot and cold sides of the TED.

A simplified steady‐state thermal resistance model is considered under specific and time‐invariant physiological, contact, and environmental conditions. By neglecting electrical power input and internal heat generation, a common heat flow is assumed across the system, such that *Q*_total_ = *Q*_TED_. By combining these equations, the effective temperature difference across the w‐TED is obtained as

(23) $$\text{Δ}T_{\mathrm{TED}}=\frac{{R^{\mathrm{th}}}_{\mathrm{TED}}}{{R^{\mathrm{th}}}_{\mathrm{TED}}+{R^{\mathrm{th}}}_{c,\mathrm{skin}}+{R^{\mathrm{th}}}_{\mathrm{TED}}+{R^{\mathrm{th}}}_{\mathrm{sink}}}\times \text{Δ}T_{\text{total}}$$

This equation indicates that a larger fraction of the available temperature difference is retained across the w‐TED when *R* ^th^_skin_, *R* ^th^_c,skin_, and *R* ^th^_sink_ are reduced relative to *R* ^th^_TED_. However, *R* ^th^_TED_ should be optimized rather than simply increased because a high internal thermal resistance can preserve the temperature difference while restricting heat flow.

On the body side, *R* ^th^_skin_ represents heat transport through the skin and the underlying tissue. Effective heat replenishment by blood perfusion lowers this resistance, whereas thick, thermally insulating tissue of restricted perfusion increases it. At the skin‐device interface, the relation *R* ^th^ = 1/*hA* shows that improved conformal contact reduces *R* ^th^_c,skin_. Contact pressure and device conformability increase the effective contact area, while curvature, movement, and loss of adhesion can form local gaps and increase the resistance. Sweat can affect both interfacial heat transfer and contact stability. However, maintaining low interfacial resistance through sustained pressure or occlusive adhesion may compromise comfort and skin compatibility during prolonged wear. On the ambient side, airflow increases the heat‐transfer coefficient and reduces *R* ^th^_sink‐amb_, whereas clothing coverage limits heat dissipation and increases *R* ^th^_sink‐amb_. Because skin contact, physiological heat transport, and ambient boundary conditions change during wear, these resistances are not fixed, and the temperature difference distributed across the w‐TED may vary accordingly.

The internal thermal resistance of w‐TED must also be considered, which consists of contributions from substrates, electrodes, thermoelectric legs, and interfacial resistances. As shown in Figure 7b, the internal thermal resistance of w‐TED can be expressed as

(24) $${R^{\mathrm{th}}}_{\mathrm{TED}}={R^{\mathrm{th}}}_{\mathrm{sub},h}+2{R^{\mathrm{th}}}_{\mathrm{el}}+\frac{{R^{\mathrm{th}}}_{\mathrm{int}}}{N\cdot A_{\mathrm{leg}}}+{R^{\mathrm{th}}}_{\mathrm{leg}}+{R^{\mathrm{th}}}_{\mathrm{sub},c}$$

where *R* ^th^_sub,h_ and *R* ^th^_sub,c_ denote the thermal resistance of the substrates on the hot and cold sides, respectively, and *R* ^th^_el_ represents the thermal resistance of the electrodes. The term *R* ^th^_leg_ indicates the thermal resistance of the thermoelectric legs, while *R* ^th^_int_ represents the effective area‐specific interfacial thermal resistance associated with the electrode‐leg and electrode‐substrate interfaces. Here, *A*_leg_ is the cross‐sectional area of an individual p‐ or n‐type leg, with p‐ and n‐type legs assumed to have equal cross‐sectional areas.

This expression highlights that interfacial thermal resistance is not merely a secondary effect but a critical component of the overall internal thermal resistance. In particular, temperature drops occurring at the interfaces reduce the effective temperature gradient delivered to the thermoelectric legs. Therefore, engineering interfacial contact to minimize thermal resistance is an essential strategy for improving the device performance.

In addition, because the p‐ and n‐type legs are thermally connected in parallel between the hot and cold sides, their equivalent thermal resistance can be expressed based on Fourier's law as

(25) $${R^{\mathrm{th}}}_{\mathrm{leg}}=\frac{h_{\mathrm{leg}}}{N\left(\lambda_{p}+\lambda_{n}\right)\cdot A_{\mathrm{leg}}}$$

where *h*_leg_ is the leg height, and *λ*_p_ and *λ*_n_ are the thermal conductivities of the p‐type and n‐type materials, respectively. This relationship indicates that *R* ^th^_leg_ is governed not only by the intrinsic material properties but also by the geometric design of the thermoelectric legs. Consequently, both material selection and structural optimization of the legs play a crucial role in controlling internal thermal resistance and enhancing thermoelectric performance.

In addition to thermal transport, electrical losses also play a critical role in determining the overall performance of w‐TEDs. Electrically, minimizing losses requires reducing both the contact resistance at the interfaces between interconnects and thermoelectric elements and the intrinsic resistance of the electrodes. The total electrical resistance of the w‐TED can be expressed as *R*_TED_ = *R*_leg _+ *R*_el _+ *R*_interface_, and associated Joule heating is given by *Q*_Joule_ = *I* ^2^ *R*_TED_. Lower interfacial resistance facilitates efficient charge carrier transport and suppresses localized Joule heating at the interfaces, thereby limiting parasitic heat generation and improving device efficiency [87, 88, 89].

Accordingly, this section provides strategies for reducing electrical contact resistance and optimizing thermal transport pathways to enhance the device performance and sustain its reliability.

### 4.1 External Thermal Management Strategies

Conventional heat sinks used in commercial products, such as refrigerators and electronic cooling systems, typically utilize metal‐based materials with fin‐shaped geometries to maximize the heat dissipation efficiency. However, these designs are not suitable for w‐TEDs, which require lightweight and stretchable components. Replacing these conventional heat sinks with soft structures can increase *R* ^th^_sink_ because of their lower thermal conductivity and less effective heat‐spreading geometries. The increase in thermal resistance hinders heat transfer from the hot side to the surrounding air and reduces the effective temperature difference sustained across w‐TED. To address this trade‐off, extensive research has focused on developing soft heat sinks with high thermal conductivity. For example, Huo et al. fabricated heat sinks by integrating a graphene composite embedded in polyurethane (PU) with paraffin (PA). Phase change materials (PCMs), such as PA, absorb a substantial amount of latent heat during the solid–liquid phase transition (Figure 8a) [90]. Through this mechanism, the system overcomes the inherent limitations of conventional thermal dissipation strategies that rely on conduction and convection. As shown in Figure 8b, the device without the PCM exhibited saturation of the cooling effect at 20 °C when the driving current exceeded 300 mA. In contrast, embedding the PCM with a melting temperature of 28 °C reduced the device temperature by 13.4 °C under 400 mA at an ambient temperature of 24 °C. However, the inherently low thermal conductivity of PCMs leads to slow heat dissipation during the phase transition process, which often requires long recovery times and specific temperature conditions for reusability. To enhance heat transfer, thermally conductive nanomaterials are commonly incorporated into PCMs. Nevertheless, this approach often suffers from aggregation of the nanomaterials during repeated thermal cycling, and the lack of strong interparticle bonding often limits the formation of continuous heat conduction pathways. To overcome these limitations, Fok et al. introduced a nanotextured Cu foam matrix by growing Cu(OH)_2_ nanowires on the Cu foam (Figure 8c), thereby forming a robust and interconnected thermally conductive network [91]. By increasing the interfacial area with the PCM, nanowires on the Cu foam enhance the heat transfer and provide abundant nucleation sites, thereby facilitating the initiation of solidification and accelerating the overall phase transition (Figure 8d). Differential scanning calorimetry (DSC) analysis shows that the nanotextured Cu foam‐PCM composite exhibits steeper thermal response curves than the conventional Cu‐PCM system, indicating faster heat absorption and release during the phase transition. Consequently, the composite achieves accelerated thermal response with enhanced phase transition kinetics and faster charge–discharge cycling. Moreover, the temperature variation remained within ±0.12 K over 50 heating‐cooling cycles, confirming excellent thermal stability under repeated thermal loading conditions (Figure 8e).

**FIGURE 8:**
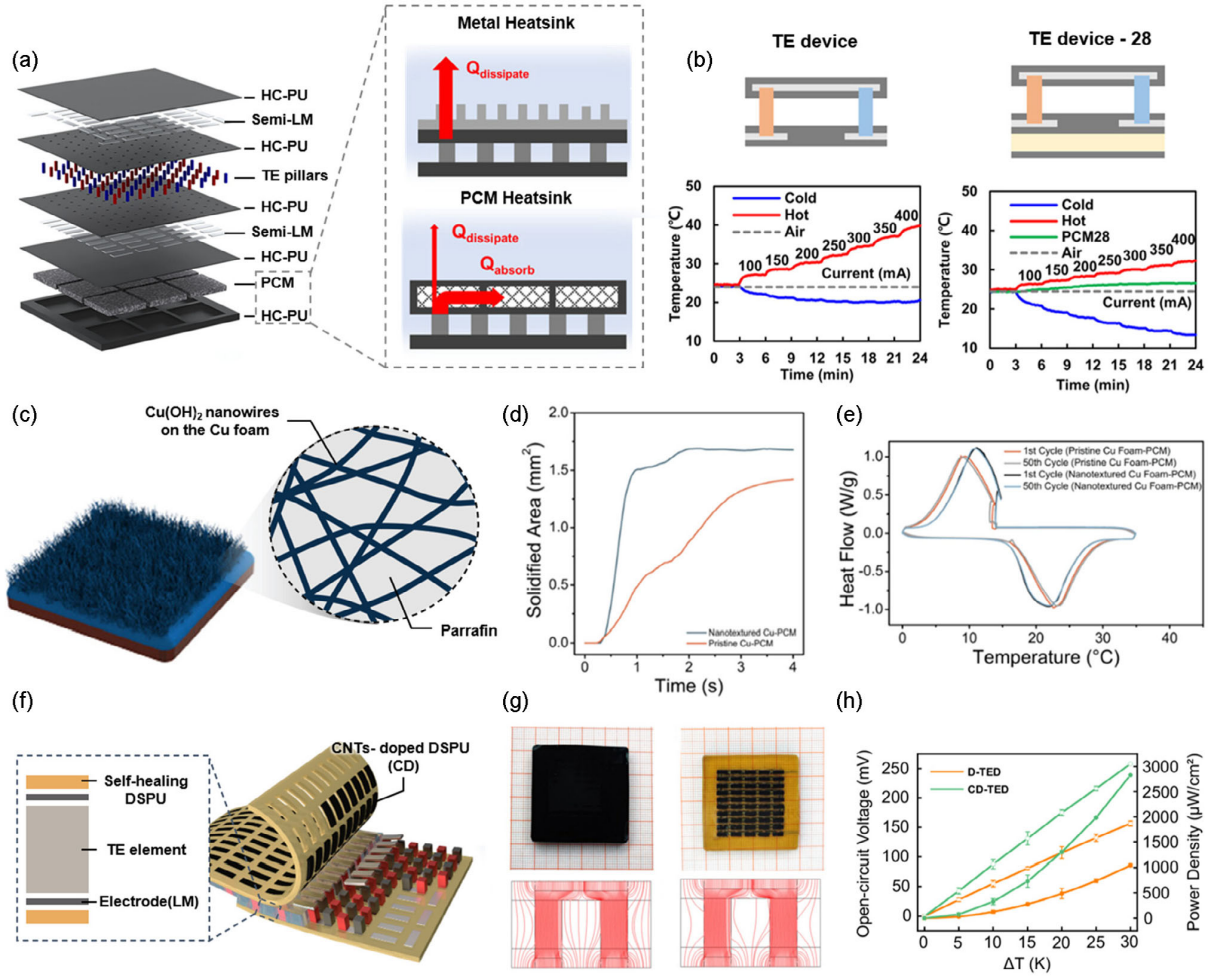
External thermal management methods. (a) Conceptual illustration of TED and comparison of working mechanisms with metal and phase change material (PCM) heat sinks. (b) Temperature response of bare TED and the TED integrated with a 28 °C melting‐point PCM under stepwise current changes at 24 °C. Reproduced with permission [90]. Copyright 2023, ACS Publications. (c) Schematic illustration of nanotextured Cu foam filled with paraffin. (d) Solidified area as a function of freezing time and (e) DSC curves of pristine and nanostructured Cu‐PCM. Reproduced with permission [91]. Copyright 2025, ACS Publications. (f) Schematic illustration of a TED incorporating EGaIn interconnects and self‐healing carbon nanotube‐doped disulfide crosslinked polyurethane (CD). (g) Optical images and finite element analysis comparing fully encapsulated and site‐selectively encapsulated devices using CNT‐doped DSPU. (h) Output performance versus temperature difference for devices with different encapsulation strategies. Reproduced under the terms of the CC‐BY 4.0 License [92]. Copyright 2025, The Authors, published by Springer Nature.

Although Cu foam with PCM‐based heat sinks enhances heat dissipation, excessive heat spreading through these materials can create parasitic thermal pathways that reduce the effective temperature difference across the thermoelectric legs. To address this issue, Sun et al. proposed a selective encapsulation strategy using self‐healable disulfide‐crosslinked polyurethane (DSPU) with different thermal conductivities (Figure 8f) [92]. CNT‐doped DSPU (CD) was applied to the LM‐based interconnects to provide higher thermal conductivity, while pristine DSPU encapsulated the surrounding regions. This design localizes heat transfer to the active regions and mitigates device performance degradation caused by parasitic heat loss (Figure 8g). As a result, the device with selective encapsulation exhibited 64% higher maximum *V*_OC_ and 171% higher maximum power density than the device fully encapsulated with DSPU (Figure 8h).

External thermal management is essential for effectively dissipating heat from the device and maintaining stable operation. PCM‐based heat sinks can temporarily absorb heat, but their finite heat storage capacity and slow regeneration limit continuous use. Integrating metal foam into PCM‐based systems or incorporating highly conductive fillers into elastomer heat sinks can improve heat dissipation. However, these approaches may increase the device weight and compromise mechanical softness. A site‐selective thermal management strategy, in which high‐performance heat dissipation components are placed only near thermoelectric legs, can reduce the overall weight and volume of the device. However, localized heat dissipation may produce nonuniform temperature distributions or shift thermal accumulation to adjacent regions. Therefore, future systems should combine lightweight and deformable materials with thermal management structures that continuously release accumulated heat to the environment and repeatedly recover their heat dissipation capacity.

### 4.2 Internal Thermal Pathway Engineering

Although considerable efforts have been devoted to improving the thermal performance of soft heat sinks, efficient heat transfer remains challenging. Accordingly, further performance enhancement requires not only ambient‐side heat transfer but also optimization of the internal thermal pathway within the w‐TED. Such optimization can be achieved by adjusting the geometry and arrangement of the thermoelectric legs or by adopting device architectures that help sustain a larger temperature difference across the thermoelectric legs. Kim et al. demonstrated this concept by systematically varying the leg length and fill factor (FF) to identify an optimized design for internal thermal transport [93]. To investigate the influence of the device geometry, devices with 31 thermoelectric leg pairs and varying leg lengths were fabricated (Figure 9a). In a separate set of devices, the leg length was fixed at 3 mm, while the number of leg pairs was varied to modulate the FF (Figure 9b). Transient analysis of these configurations enabled the identification of the optimal thermoelectric leg length and FF. As shown in Figure 9c, increasing the leg length enhances the thermal resistance of the thermoelectric elements, thereby inducing a larger temperature difference across the legs and leading to an increased device performance. However, when the leg length exceeds 1.5 mm, the output voltage begins to decrease because of the increased internal electrical resistance associated with longer legs. A similar trade‐off is observed for FF (Figure 9d). As the FF increases, the active thermal pathway through the thermoelectric legs becomes more dominant. However, overly large FF values increase the overall thermal conductance of the device, making it difficult to sustain a stable temperature difference. Conversely, when the FF is small, the proportion of nonactive regions between the legs increases, promoting heat transfer through parasitic bypass pathways and reducing the effective temperature drop across the thermoelectric elements. The transient simulation results are consistent with experimental observations, confirming the existence of optimal geometric parameters for effective internal thermal pathway engineering.

**FIGURE 9:**
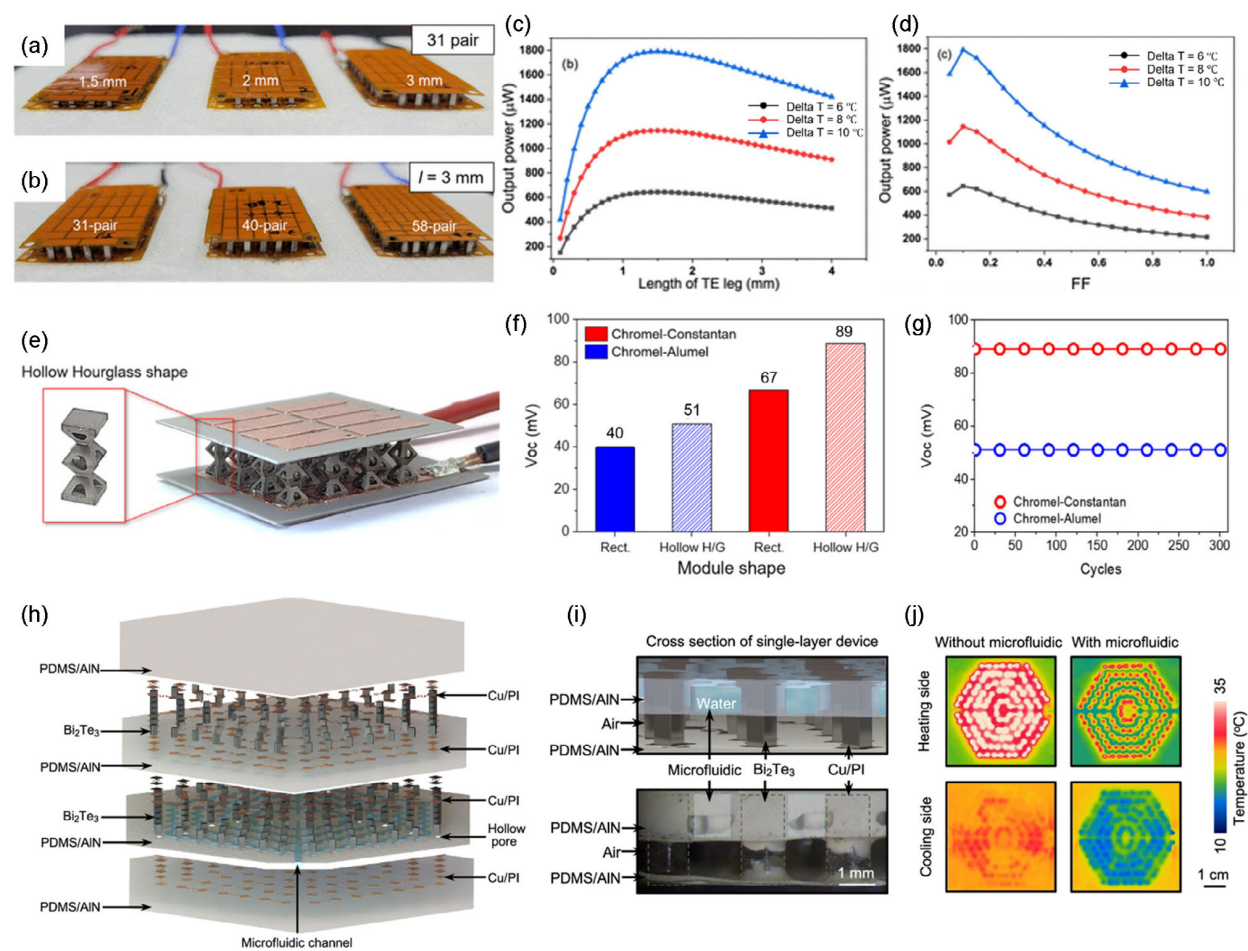
Internal thermal management methods. (a,b) Optical images of TEDs with different leg lengths and p–n pairs. (c, d) Power as a function of thermoelectric leg length and fill factor under different temperature differences. Reproduced under the terms of the CC‐BY 4.0 License [93]. Copyright 2024, The Authors, ACS Publications. (e) Photographs of the TED with hollow hourglass‐shaped legs. (f,g) *V*_OC_ as a function of leg material and geometry, and as a function of thermal cycles. Reproduced with permission [94]. Copyright 2025, ACS Publications. (h) Schematic illustration of the TED with microfluidic channels for efficient heat management and (i) Cross‐sectional image showing microfluidic channels and the upper halves of the thermoelectric legs surrounded by the microfluidic channels. (j) Thermal imaging of the device with and without microfluidic channels. Reproduced with permission [95]. Copyright 2025, The Authors, published by Springer Nature.

Beyond optimizing the leg length and FF, further improvements can be achieved by tailoring the geometry of individual thermoelectric legs. For example, Fabián‐Mijangos et al. fabricated pyramidal thermoelectric legs that reduced the overall thermal conductance of the device by varying the cross‐sectional area along the leg height [96]. Because thermal conductance is proportional to the cross‐sectional area, locally reducing the area lowers the thermal conductance. Therefore, placing a narrower cross‐sectional region on the hot side increases the local thermal resistance of the leg, thereby limiting conductive heat transfer and enhancing the temperature difference across the thermoelectric legs. These results suggest that the leg geometry can serve as an effective design parameter for thermal management. However, in conventional TEDs, geometric modifications have generally been limited to relatively simple forms, such as hourglass (H/G) or trapezoidal geometries, because the brittleness of most thermoelectric materials constrains the fabrication of more complex structures. In contrast, metal‐based thermoelectric elements offer greater geometric flexibility, enabling more sophisticated leg designs that further increase thermal resistance and improve device performance. Koo et al. fabricated an 8‐pair w‐TED using p‐type Ni_90_Cr_10_ paired with either n‐type Cu_55_Ni_45_ or Ni_95_Al_2_Mn_2_Si_1_ as the n‐type material (Figure 9e) [94]. Using chromel–constantan (C–C) and chromel‐alumel (C–A) material pairs, the authors constructed the device with conventional rectangular legs or hollow H/G‐shaped legs for comparison. Under a fixed temperature condition where the hot and cold sides were maintained at 473 and 323 K, respectively, the hollow H/G module employing the C–C pair generated the highest *V*_OC_ of approximately 89 mV (Figure 9f). This performance enhancement was attributed to the hollow H/G geometry, which increased the thermal resistance of the thermoelectric legs and consequently amplified the internal temperature difference across the device. Also, by alternating the hot‐side temperature between 323 and 473 K for a total of 300 cycles, the module maintained a stable *V*_OC_ (Figure 9g). These results show that this geometry exhibits excellent electrical and thermal stability under repeated thermal stress conditions.

In addition to the thermoelectric‐leg design, internal thermal transport can also be tailored through device architecture, as demonstrated by Liu et al. [97]. By stacking optimized legs in two layers rather than extending a single leg length, a larger temperature difference can be sustained across the device, resulting in improved thermoelectric performance. Based on this double‐layer architecture, Huang et al. incorporated microfluidic channels between device layers to further optimize thermal management (Figure 9h) [95]. The upper half of the thermoelectric legs was embedded along the sidewalls of the microfluidic channels, which increased the contact area between the legs and the channels, leading to enhanced cooling performance (Figure 9i). A direct comparison of device operation with and without microfluidic channels revealed a distinct thermal behavior, as shown in Figure 9j. In the absence of the channels, insufficient heat dissipation caused the hot‐side temperature to exceed 45 °C, while heat transfer from the hot side to the cold side led to a rapid increase in the cold‐side temperature beyond 26 °C, surpassing the ambient temperature. Furthermore, even after the device was turned off, the residual heat stored within the device continued to propagate, maintaining the cooling side at a temperature higher than that of the ambient. In contrast, the device incorporating microfluidic channels exhibited efficient heat release, maintaining stable temperatures of 30 °C on the hot side and 20.6 °C on the cold side. After the device was switched off, both regions rapidly returned to their initial temperatures, demonstrating the effectiveness of microfluidic channels in the internal thermal regulation.

Internal pathway engineering is essential for improving both the electrical and thermal performance of w‐TEDs. However, modifying the thermoelectric leg geometry or packing density involves trade‐offs between electrical and thermal resistance. Multilayers can further improve the device performance, but they may increase thickness, interfacial resistance, and fabrication complexity. Moreover, these strategies have mainly been applied to rigid thermoelectric legs and may be difficult to implement with deformable thermoelectric materials, which are more suitable for w‐TEDs. Accordingly, future research should focus on material‐level strategies, such as controlled doping and microstructural optimization, that simultaneously enhance electrical transport without relying on specific leg geometries or complex structural designs.

### 4.3 Strategies to Reduce Electrical Contact Resistance

When thermoelectric materials are integrated with metal electrodes such as Ag or Cu, interdiffusion can occur, particularly during prolonged or elevated‐temperature operation. This process leads to the formation of intermetallic compounds (IMCs) at the interface [98]. The resulting IMCs increase both electrical and thermal contact resistance and, due to their intrinsic brittleness, can induce cracking. Ultimately, this results in mechanical failure and device degradation. To suppress this elemental migration, various metal and metal alloy diffusion barriers, such as Ni, Cr, Ti, and Mo, are inserted between the thermoelectric elements and the electrodes [99, 100, 101, 102, 103]. Among these, Ni is widely utilized for Bi_2_Te_3_‐based thermoelectric materials due to its excellent mechanical compatibility, with a coefficient of thermal expansion (CTE) closely matched to that of the Bi_2_Te_3_ material family. Its superior wettability and spreadability with solder also make it an ideal diffusion barrier for suppressing atomic interdiffusion between Bi_2_Te_3_‐based thermoelectric materials and the solder [104]. However, a Ni diffusion barrier alone is insufficient at elevated temperatures as its reaction with thermoelectric materials promotes brittle IMC growth, leading to increased contact resistance and greater interfacial stress. To address this issue, Sun et al. introduced a Ni/Ti bilayer, where Ti possesses a CTE similar to that of Bi_2_Te_3_, to ensure stability above 473 K (Figure 10a) [105]. The formation of the TiTe_2_ reaction layer ensures strong metallurgical bonding, while the loose structure of the Ti layer reduces its Young's modulus and enables particle sliding. These combined effects effectively release interfacial shear and tensile stresses arising from the thermal mismatch. This configuration facilitates Ohmic contact, resulting in a low contact resistivity of 2.5 μΩ cm^2^ for p‐type and 2.3 μΩ cm^2^ for n‐type materials, respectively (Figure 10b). Park et al. employed an Fe‐Ni–Cr diffusion barrier to suppress Ni migration in a temperature range from 200 to 250 °C (473.15–523.15 K) [108]. This approach produced thinner IMCs compared to pure Ni barriers, maintaining a contact resistance of approximately 10^−5^ Ω cm^2^ for n‐type legs even after 100 h of exposure at 250 °C.

**FIGURE 10:**
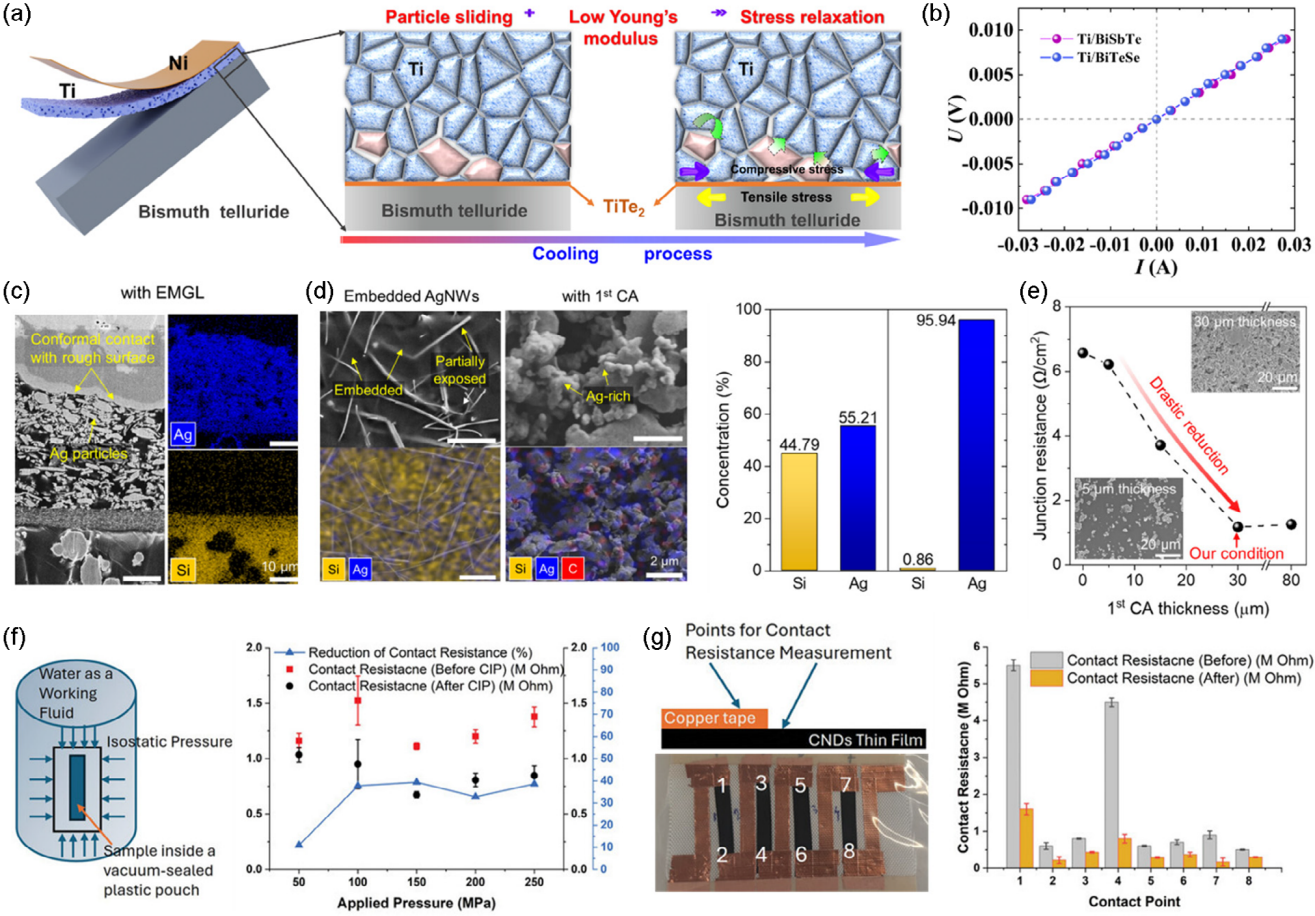
Strategies for reducing electrical contact resistance. (a) Schematic diagram of interfacial stress release mechanism by a lowered Young's modulus and particle sliding. (b) *I*–*V* characteristics of the Ti/BiSbTe and Ti/BiTeSe junctions, showing ohmic contact. Reproduced under the terms of the CC‐BY 4.0 License [105]. Copyright 2023, The Authors, published by Springer Nature. (c) Scanning electron microscopy (SEM) and energy dispersive spectroscopy (EDS) mapping images of the electromechanically graded interlayer (EMGL) to ensure a stable interface between rigid thermoelectric elements and soft interconnects. (d) SEM and EDS mapping images of embedded AgNWs within the soft interconnects and of the first conductive adhesive (CA), with Si/Ag ratio comparison before and after EMGL application. (e) Junction resistance between the thermoelectric leg and soft interconnect as a function of the first CA thickness. Reproduced with permission [106]. Copyright 2023, ACS Publications. (f) Schematic of cold isostatic pressing setup and contact resistance of five different samples before and after application of different pressures. (g) Photograph of the thermoelectric device comprising carbon nanodots (CNDs) and copper electrodes, along with contact resistance at each point before and after applying pressure. Reproduced with permission [107]. Copyright 2025, The Authors, published by Wiley‐VCH GmbH.

As w‐TED designs incorporate soft electrodes and interconnects, *R*_el_ and *R*_interface_ can increase because these compliant conductors generally exhibit lower electrical conductivity than rigid metals and may undergo resistance changes, interfacial debonding, or contact degradation under repeated deformation. This has motivated a broader set of interface strategies that simultaneously improve chemical stability and mechanical robustness under repeated deformation. One representative strategy for soft interconnect integration is exemplified by the work of Cho et al. [106], who introduced a novel type of conductive adhesive (CA) termed an electromechanically graded interlayer (EMGL) between Ni‐treated Bi_2_Te_3_ and Ag nanowire (AgNW)‐based elastic conductor, as shown in Figure 10c. In AgNW‐based elastic conductors, conductive particles are embedded within the elastomer matrix, which limits the number of exposed contact points and consequently leads to a higher contact resistance. The first CA layer in the EMGL is designed as an Ag‐rich layer, increasing the Ag concentration at the interface from 55.21% to 95.94%, thereby effectively reducing the contact resistance (Figure 10d). The thickness of this layer was optimized to 30 μm to minimize the contact resistance and maximize the mechanical bonding strength (Figure 10e). Subsequently, the second CA of EMGL serves as a chemical and mechanical buffer, suppressing Ag diffusion to prevent brittle IMC formation. It also ensures robust contact by conformally filling the gaps between the rough thermoelectric surface and the first layer. For compliant OTE materials, minimizing contact resistance becomes more critical due to their inherently low thermoelectric performance. Ahmed et al. illustrated the reduction of contact resistance between the carbon nanodot (CND)‐based thermoelectric film and the Cu electrode through cold isostatic pressing (CIP) (Figure 10f) [107]. Upon application of 150 MPa, the film thickness was reduced by 20%, and the resistance was decreased to 0.6 MΩ, corresponding to a 40% reduction compared to that in the initial state. This improvement primarily originates from pressure‐induced densification at the interface between the CND film and the Cu electrode. This result was confirmed through measurements at multiple points as demonstrated in Figure 10g. In addition, Hwang et al. introduced a polystyrene (PS) layer to decrease contact resistivity between PEDOT:PSS thermoelectric layers and Ag‐conductive inks [109]. In the absence of the PS layer, the contact resistivity was measured to be 6.02 × 10^−2^ Ω cm^2^ at 300 K and exhibited temperature dependence, indicating thermionic emission‐dominated transport associated with a Schottky barrier. In contrast, by adding a PS layer that shifts the main electron injection mechanism to hopping‐assisted tunneling, a contact resistivity of 2.77 × 10^−2^ Ω cm^2^ was maintained across a wide temperature range without significant variations.

Contact resistance engineering is important for w‐TEDs because even modest interfacial resistance reduces the output power or increases Joule heating. Barrier layers can suppress interfacial diffusion and undesirable reactions, thereby maintaining low contact resistance, but they require additional deposition steps and may introduce mechanically rigid layers. Pressure‐assisted contact can reduce contact resistance but requires additional components for applying pressure, which complicates fabrication. Moreover, nonuniform or excessive pressure may cause variations in contact resistance or mechanical damage to the thermoelectric elements and interfaces. Graded interfaces appear particularly promising because they mitigate an abrupt material mismatch across the interface. However, most contact resistance studies have focused on rigid thermoelectric elements, while interfaces involving deformable thermoelectric materials remain insufficiently investigated. Future research should establish interface engineering strategies that maintain low contact resistance and stable electrical contact under repeated deformation across rigid, flexible, and stretchable thermoelectric leg configurations.

## 5 Applications and Practical Considerations

When externally powered, w‐TEDs can operate in the active thermal regulation mode for both personal comfort and temperature‐sensitive components within wearable systems. Conversely, in the passive power generation mode, these devices can efficiently convert metabolic heat dissipated from the human body into electrical energy, enabling them to serve as self‐sustaining power sources for the continuous operation of wearable devices. Beyond energy conversion, the responsive electrical output of w‐TEDs can be utilized as functional signals for various biophysical and environmental sensing applications. In this section, we review the diverse applications of w‐TEDs across these operational modes while also considering the system‐level constraints and reliability requirements that govern their practical translation.

### 5.1 Thermal Management

When operated as a thermoelectric cooler (TEC), a w‐TED actively pumps heat under an applied current through the Peltier effect. For PTM, the cold side is placed in contact with the skin to remove heat toward the ambient side. As shown in Figure 11a, a TEC composed of 143 pairs of thermoelectric legs was integrated into a headband with an LM skeleton‐based active cooling elastomer, enabling precise thermoregulation of the forehead under various environmental conditions [110]. When a direct current is applied, the cold side of the TEC absorbs heat from the forehead and transfers it to the hot side. The device incorporates a microcontroller unit and a proportional integral derivative (PID) controller to dynamically regulate the temperature. The forehead temperature measured by an integrated sensor was transmitted to a mobile application via Bluetooth, allowing real‐time monitoring and adjustment of the target temperature. By controlling the magnitude and direction of the applied current, the system can achieve either cooling or heating (Figure 11b). Even when the ambient temperature varies significantly from 20 to 42 °C, the forehead temperature can be maintained at a preset value of 32 °C with a fluctuation of only ±0.25 K (Figure 11c).

**FIGURE 11:**
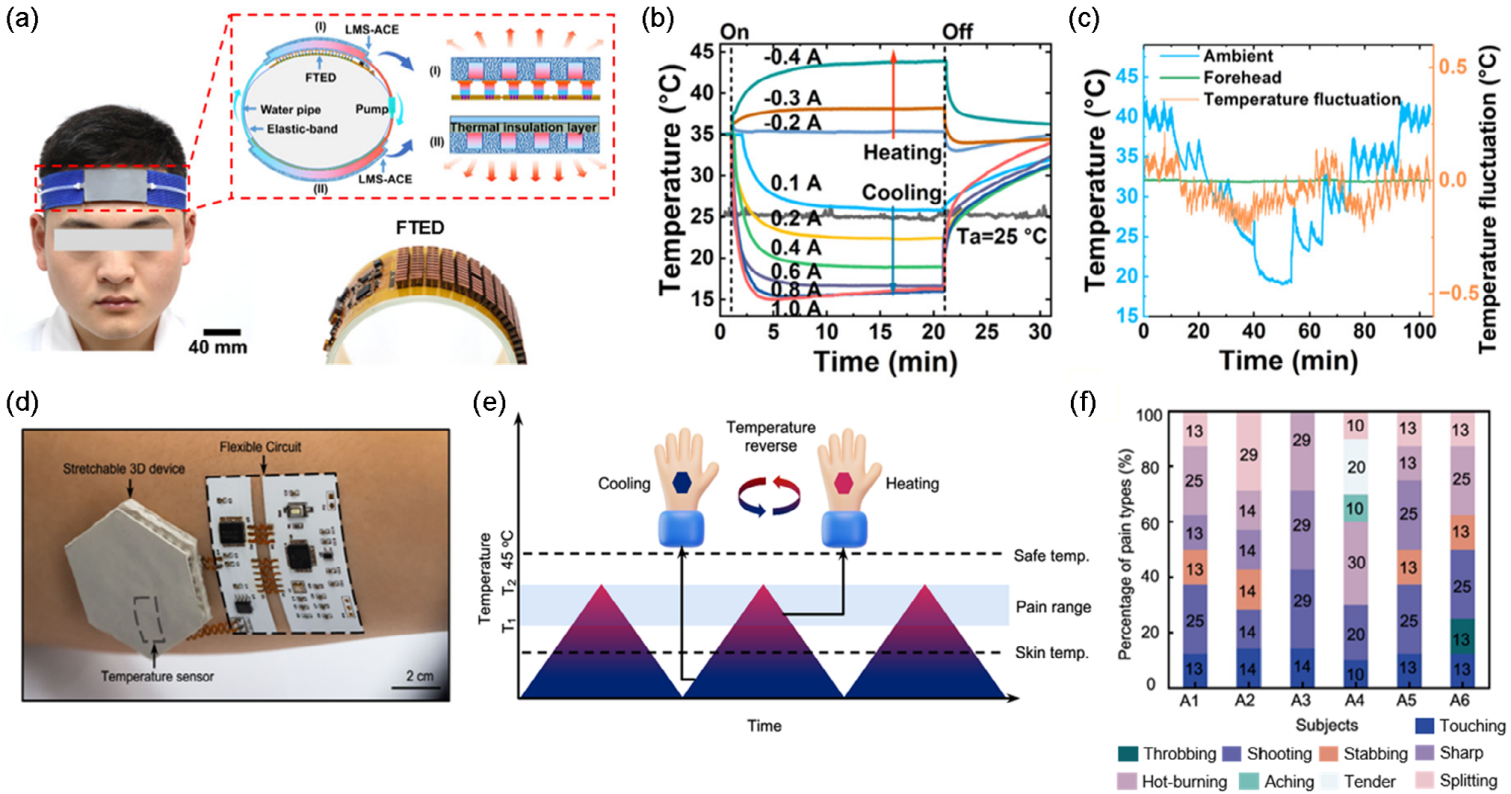
Adaptive thermoregulation across diverse environments utilizing w‐TEDs. (a) Optical image of the cooling band integrated with a TED and the illustrations of the working principle. (b) Cooling and heating performance of the TED regulated by Proportional‐Integral‐Derivative (PID) controller. (c) Temperature regulation performance of the device under severe temperature fluctuations. Adapted under the terms of the CC‐BY 4.0 License [110]. Copyright 2024, The Authors, published by Springer Nature. (d) Photographs of the closed‐loop system attached to skin. (e) Illustration of the principle of thermal grill illusion and (f) different types of virtual pain induced by the system. Adapted with permission [95]. Copyright 2025, The Authors, published Springer Nature.

The thermoregulation capability of w‐TEDs can also be utilized in virtual reality and augmented reality (VR/AR) technologies to provide realistic thermal feedback through rapid temperature modulation. Hung et al. presented a multilayer structure and microfluidic channels to achieve superior thermal management, enabling a fast temporal response and rapid bidirectional thermal switching [95]. The fabricated device was integrated with the flexible printed circuit board (FPCB) and mounted on the forearm, as illustrated in Figure 11d. By alternating between hot and cold stimuli at high speeds, the system induced paradoxical pain perception. Fluctuating temperatures within the 25–40 °C range could generate significant pain sensations without causing any damage to the skin (Figure 11e). The intensity of the perceived pain can be modulated by adjusting the magnitude, speed, and frequency of the temperature shift. In experiments with 6 subjects, they felt intense sensations, such as stabbing or shooting pain (Figure 11f). The initial human evaluation supports the feasibility of controllable thermal feedback, while broader user studies would help establish consistent perceptual responses across individuals and operating conditions.

Despite these demonstrations, power source dependence remains a key barrier to the practical use of TEC‐based PTM. Increasing battery capacity extends the operating time but adds rigid volume and weight, thereby compromising wearability. In the LM skeleton‐based active cooling elastomer (LMS‐ACE) headband, both the TEC and water pump required power, yet the reported device weight excluded the battery, and the 24 h test was conducted under a stable power supply. By contrast, the VR/AR thermal‐feedback device developed by Huang et al. provided a complete power budget, but its 3.7 V, 600 mAh battery supported only approximately 1.9–2 h of operation. Therefore, practical TEC‐based PTM requires lower power consumption through optimized TEC arrays, more efficient driving circuits, and low‐power auxiliary components. This would reduce the required battery capacity while improving safety, comfort, and long‐term wearability.

While these applications focus on direct thermal regulation of the wearer, the same active heat‐pumping principle can also be applied to thermal control within the wearable system itself. Battery thermal management (BTM) is one potential application, particularly as wearable electronics increasingly integrate batteries into compact platforms with limited space for heat dissipation. Maintaining an appropriate battery temperature is important not only for reliable operation but also for user safety because these devices are worn in close proximity to the body. TEC‐based BTM has been investigated in conventional battery systems using combinations with air or liquid cooling, phase‐change materials, and heat pipes [111]. Its application to wearable electronics, however, remains relatively unexplored. Developing TEC‐based BTM without substantially increasing the form factor or power burden of the overall device could therefore provide a wearable‐friendly approach to battery temperature regulation.

### 5.2 Thermoelectric Generator (TEG)

When operating in the power generation mode based on the Seebeck effect, TEDs are commonly referred to as thermoelectric generators (TEGs). To demonstrate their capability as a primary power source, Lee et al. developed a soft TEG comprising 200 p–n pairs and connected to an FPCB to drive a functional system consisting of a voltage converter and light‐emitting diodes (LEDs) for a temperature‐detectable oven glove (Figure 12a) [112]. By introducing this device, the researchers achieved efficient energy harvesting from arbitrarily shaped heat sources, generating an output voltage of 0.56 V. This energy was subsequently boosted to 1.66 V by a voltage converter, providing sufficient electrical power to illuminate the LED (Figure 12b). Due to its mechanical flexibility, the TEG maintained conformal contact with the curved surface of a hot bottle and successfully powered the “H”‐shaped LEDs without any assistance from an external power supply, as shown in Figure 12c. Although well suited to temperature‐warning applications, this demonstration relies on the large thermal gradient of a hot object. Its relevance to the continuous self‐powered operation of wearable electronics using body heat is therefore limited.

**FIGURE 12:**
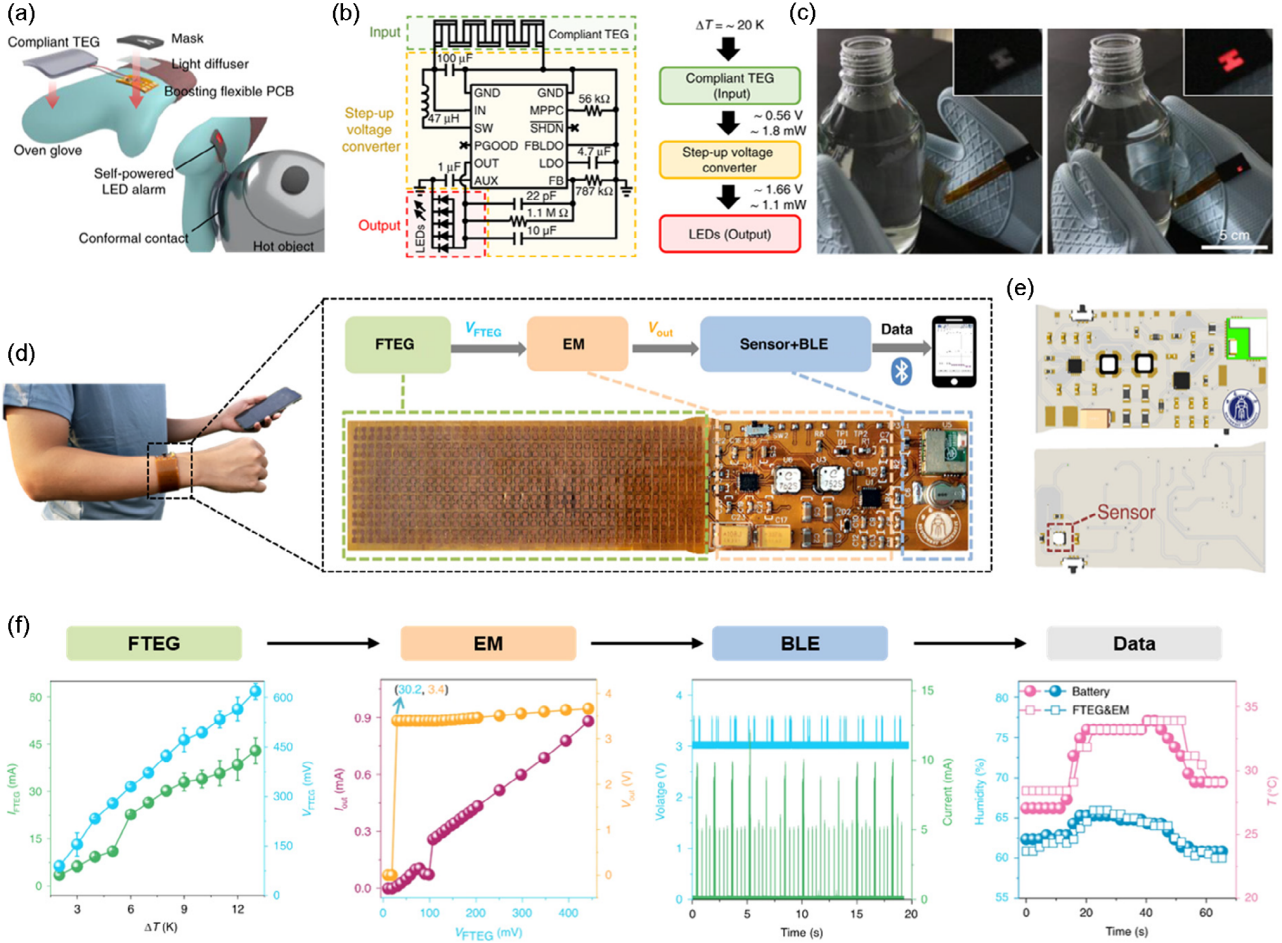
w‐TEG for self‐powering in wearable devices. (a) Schematic illustration of a hot‐surface warning glove integrated with self‐powered a light‐emitting diode (LED) system. (b) Circuit diagram and corresponding block diagram elucidating operating sequence of the system. (c) Photographs showing self‐powered system before and after contact with hot water bottle, with magnified image showing a “H” sign. Reproduced under the terms of the CC‐BY 4.0 License [112]. Copyright 2020, The Authors, published by Springer Nature. (d) Optical images of the Flexible Thermoelectric generator (FTEG) worn on wrist, along with circuit diagram and photo of self‐powered integrated circuits. (e) Schematic diagram of the flexible energy management (EM) electronics, sensors, and Bluetooth modules. (f) Output performance of FTEG, EM, Bluetooth low energy (BLE), and sensors. Real‐time data of body temperature and humidity obtained from FTEG‐powered system were comparable to those obtained from the lithium‐battery‐powered system. Reproduced under the terms of the CC‐BY 4.0 License [113]. Copyright 2023, The Authors, published by Springer Nature.

To demonstrate power generation under the smaller temperature gradients available from the human body, Yang et al. developed an autonomous sensing platform in which a high‐performance TEG served as the primary energy source for energy management (EM) electronics, a Bluetooth Low Energy (BLE) chipset, and a multimodal sensor suite (Figure 12d,e) [113]. To ensure stable power generation under the mechanical stresses induced by body motion during wear, the researchers implemented a stress‐mitigating architecture by aligning the electrode perpendicularly to the direction of the applied bending force. This optimization allowed the TEG to yield approximately 89 mV even at a minimal Δ*T* of 2 K, exceeding the 30.2 mV threshold voltage required to activate the EM circuit. By adopting a polarity‐matching topology, the EM system ensures consistent power delivery and stable capacitor charging, regardless of environmental fluctuations. The sensor and BLE module powered by this integrated system exhibited performance comparable to lithium‐ion battery‐powered systems, demonstrating the feasibility of wireless power supplied by human body heat (Figure 12f). This system demonstrates that sensing and BLE transmission are possible even at low Δ*T*, although the FPCB‐based integration includes rigid components and shows reduced output under bending. Improving conformability and deformation‐stable power generation will therefore be important for continuous on‐body use.

### 5.3 Interdisciplinary Applications

#### 5.3.1 Biophysical Sensing

The electrical power generated by TEGs can be directly used as sensing signals to detect various physiological and physical stimuli without requiring external power. He et al. developed a thermoplastic polyurethane (TPU)/CNTs fabric‐based TEG with an *S* of 49 μV K^−1^, capable of stable biomonitoring even in a humid environment (Figure 13a) [114]. When hot water was applied to the fabric, the voltage rapidly increased and gradually decayed as the water cooled. This behavior demonstrates its capability as a robust, self‐powered temperature sensor that operates effectively even under moist conditions (Figure 13b). By placing one end of the fabric in contact with the upper lip while leaving the other end exposed to the ambient air, the device directly converts the temperature change created by inhalation and exhalation into real‐time voltage signals. As shown in Figure 13c, the magnitude and rate of the output voltage fluctuate according to different breathing patterns, enabling detection of various physiological conditions, including verification of cardiopulmonary resuscitation (CPR) effectiveness through respiratory monitoring without an external power supply.

**FIGURE 13:**
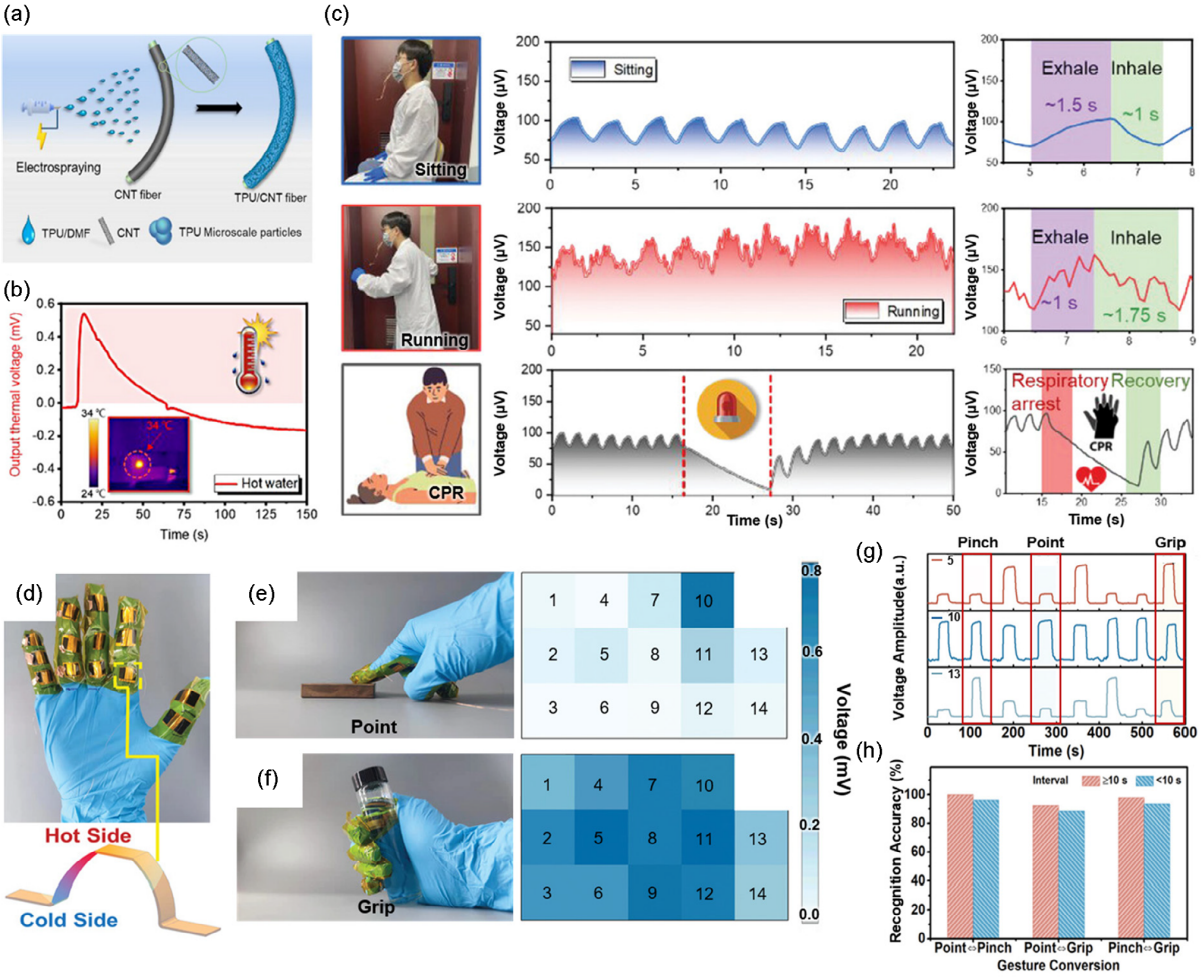
Thermoelectric sensors for physiological monitoring and gesture recognition. (a) Schematic illustrations of the fabrication process of stretchable thermoelectric fabric composed of thermoplastic polyurethane/carbon nanotube (TPU/CNT). (b) Output voltage generated upon dropping hot water onto the end of the sensor. (c) Output voltage of stretchable thermoelectric fabric integrated into a mask worn by a subject during sitting, running, and cardiopulmonary resuscitation (CPR). Adapted under the terms of the CC‐BY 4.0 License [114]. Copyright 2023, The Authors, published by John Wiley and Sons. (d) Thermoelectric sensors integrated into a glove and their schematic diagram. (e,f) Optical images of pointing and gripping gestures and corresponding voltage mapping in 14 thermoelectric sensors. (g) Voltage signals from 5, 10, and 13 sensors for distinguishing different hand gestures. (h) Recognition accuracy under randomly changing hand motions. Adapted with permission [115]. Copyright 2022, Wiley‐VCH GmbH.

TEGs that function as active sensory receptors by converting temperature differences directly into voltage signals are particularly valuable for robotics and HMIs, where eliminating external power supplies simplifies system integration [116, 117, 118]. In contrast, traditional sensing systems often face constraints due to the bulkiness of external power supplies and the complexity of signal processing [119]. The integration of TEGs enables subtle temperature differences generated during contact between the robotic e‐skin and external objects to be instantaneously converted into electrical signals. Du et al. developed a TEG‐based smart glove system using reduced graphene oxide/reduced poly(3,4‐ethylenedioxythiophene):poly(styrene sulfonate) (rGO/rPEDOT:PSS) composite to accurately identify various hand gestures [115]. A total of 14 sensor units, each consisting of an active thermoelectric composite layer sandwiched between two copper electrodes, were strategically integrated into a nitrile glove, as shown in Figure 13d. Each sensor generates a distinct Seebeck voltage signal derived from the temperature difference between the internal body temperature and the external object, enabling precise recognition of distinct hand gestures (Figure 13e,f). The system could clearly distinguish complex gestures such as pinching, pointing, and gripping by analyzing voltage outputs from only three specific sensors (5, 10, and 13), demonstrating that sophisticated recognition is achievable with a minimal sensory array (Figure 13g). Even in dynamic environments with movement, the system maintained a remarkably high recognition accuracy ranging from 88.5% to 100%, confirming its high reliability and sensing performance (Figure 13h).

For thermoelectric sensors, practical reliability depends on whether the measured signal can be attributed specifically to the intended thermal stimulus. Ambient temperature, airflow, contact pressure, and mechanical deformation can alter the temperature gradient, contact area, or electrical connection, and their effects should be distinguished from the thermoelectric response. Although the studies discussed above evaluated mechanical and environmental stability, further validation under simultaneous variations in these factors is needed to establish signal specificity and reproducibility in practical use.

#### 5.3.2 Wound Healing

Electrical stimulation supplied by TEGs can accelerate wound healing by promoting cellular migration and proliferation. Zhang et al. demonstrated that a film‐type TEG could stimulate the mechanosensitive Piezo1 ion channel [120]. This induces Ca^2+^ influx and subsequent intracellular signaling, leading to the upregulation of critical healing factors such as hypoxia‐inducible factor‐1α (HIF‐1α) and vascular endothelial growth factor (VEGF) (Figure 14a). By delivering an output of 6.4 mV at Δ*T* = 10 K, the TEG‐treated group achieved complete wound closure within 12 days, 4 days faster than that of the sham and control groups (Figure 14b). Beyond electrical stimulation alone, recent studies have developed multifunctional TEG‐based platforms incorporating antibacterial agents, such as tannic acid (TA), to effectively treat infected wounds. Gao et al. developed a TA‐containing thermoelectric hydrogel that operates based on the Soret effect (Figure 14c) [121]. In this ionic thermoelectric system, a temperature difference drives the migration of ions according to their mass and charge distribution, generating an electric field analogous to that generated by the conventional Seebeck effect. Hydrogels with different TA contents were denoted as PAAT_n_, and the reactive oxygen species (ROS) scavenging ratio increased with increasing TA concentration (Figure 14d,e). In in vivo wound healing experiments, the device containing 2 mg mL^−1^ of TA exhibited the highest collagen deposition and microvessel density. The combination of electrical stimulation with TA‐mediated anti‐inflammatory activity synergistically accelerated tissue regeneration (Figure 14f–h). These TEG‐based approaches demonstrate promising wound‐healing outcomes, although their reproducibility depends on the thermoelectric stimulus generated under changing wound and thermal conditions. As wound temperature and tissue properties evolve during healing, the temperature gradient and resulting electrical output may vary over time and among subjects. Evaluating these changes across different healing stages and wound conditions will therefore be important for establishing reproducible therapeutic efficacy.

**FIGURE 14:**
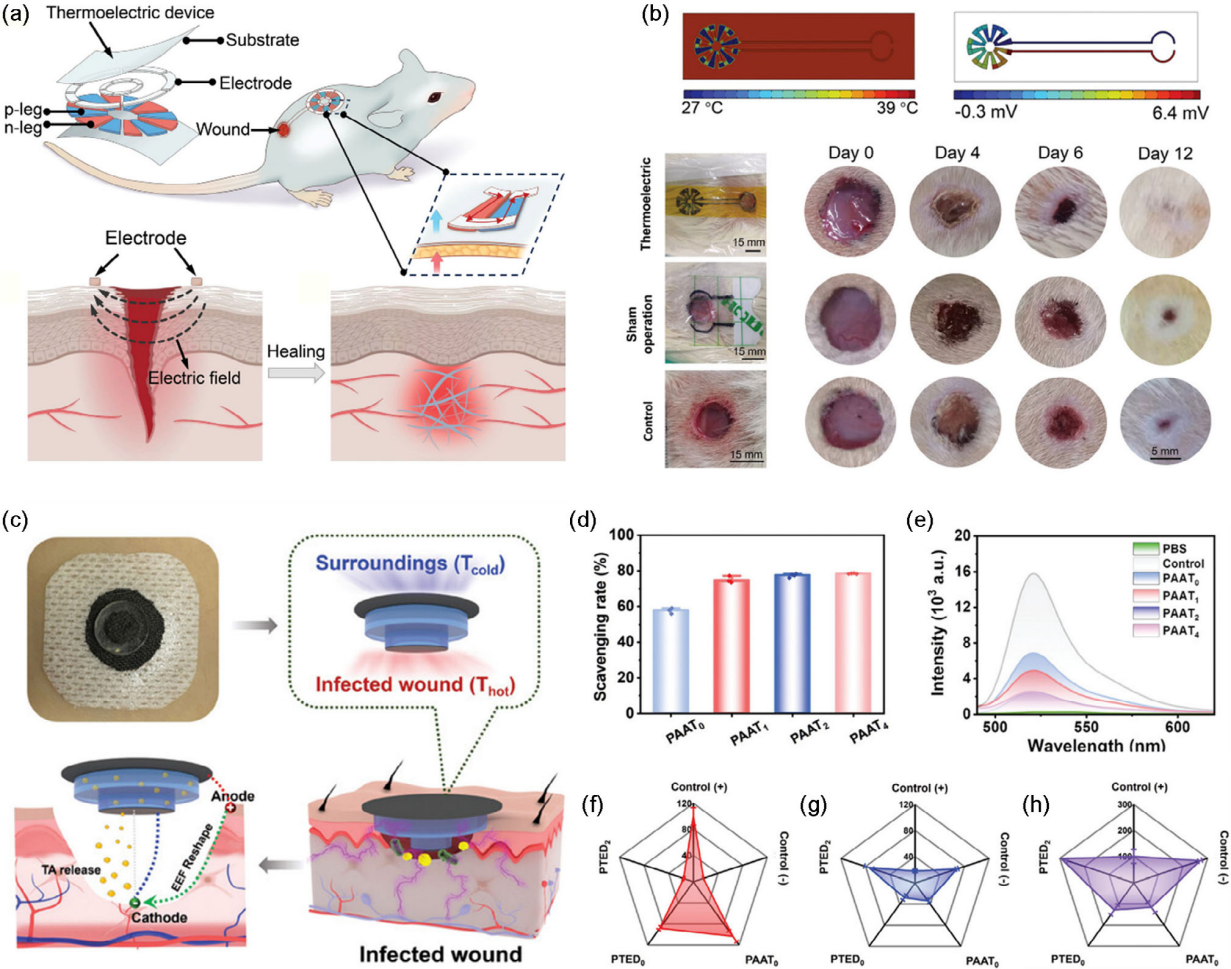
Biomedical therapeutic applications of w‐TEDs. (a) Schematic illustration of w‐TED designed to accelerate skin wound healing. (b) Finite element analysis (FEA) results showing temperature and corresponding electric field distribution, along with in vivo experiment results demonstrating accelerated wound healing process using w‐TED. Adapted with permission [120]. Copyright 2024, Wiley‐VCH GmbH. (c) Optical image of thermoelectric dressing including tannic acid (TA) for infected wound. (d) Scavenging rate of reactive oxygen species (ROS) and (e) fluorescence curve for detecting ROS. (f–h) Statistical analysis of inflammatory cells, collagen deposition, and microvessel densities. Reproduced with permission [121]. Copyright 2024, Wiley‐VCH GmbH.

Maintaining the wound site within an optimal temperature range can enhance local blood flow and tissue metabolism, thereby promoting re‐epithelialization, angiogenesis, and wound healing [122]. These findings highlight the potential of thermoelectric platforms as multifunctional therapeutic systems that combine electrical, chemical, and thermal stimulation for comprehensive wound management. More broadly, w‐TEGs have been utilized across various biomedical applications, including diagnostic sensing [123, 124] and thermal neuromodulation [125, 126].

#### 5.3.3 Emerging Applications

The proliferation of infrared (IR) surveillance technologies has led to a growing demand for active thermal camouflage systems. To address this need, Hong et al. developed a camouflage cloth by integrating thermoelectric modules into the cloth, enabling suppression of metabolic heat generated by the human body (Figure 15a,b) [127]. The camouflage performance of the TED was compared with that of a passive low‐emissivity (low‐e) Al film (*ε* = 0.04), which is widely used for conventional thermal camouflage. Although the low‐e film can conceal thermal dissipation by reflecting the surrounding thermal radiation, its camouflage capability is limited under varying background temperature conditions. In contrast, the TED‐based system actively regulates its surface temperature through thermoelectric cooling and heating, allowing the device temperature to match the surrounding background temperature. As a result, it maintained effective thermal camouflage regardless of background temperature variations (Figure 15c–f). Furthermore, a PCM layer was introduced between the TED and the cloth to absorb heat through latent heat storage. This design enabled the device temperature to be maintained for approximately 40 min, thereby sustaining the camouflage functionality over an extended period (Figure 15g). More recently, Hou et al. developed a flexible thermoelectric camouflage system that integrates IR concealment, deception, and information display within a single platform [129]. The independently controlled thermoelectric pixel array enabled spatial regulation of surface temperature, allowing the device to match the thermal background, alter the apparent temperature and shape of concealed objects, and display predefined information detectable by an IR camera. A porous black ethylene‐vinyl acetate (EVA) film layer on the top of the device improved thermal uniformity and reduced IR reflection, while kirigami enhanced bendability for curved and wearable applications. Therefore, this work extends thermoelectric camouflage from simple concealment to multifunctional thermal deception and communication.

**FIGURE 15:**
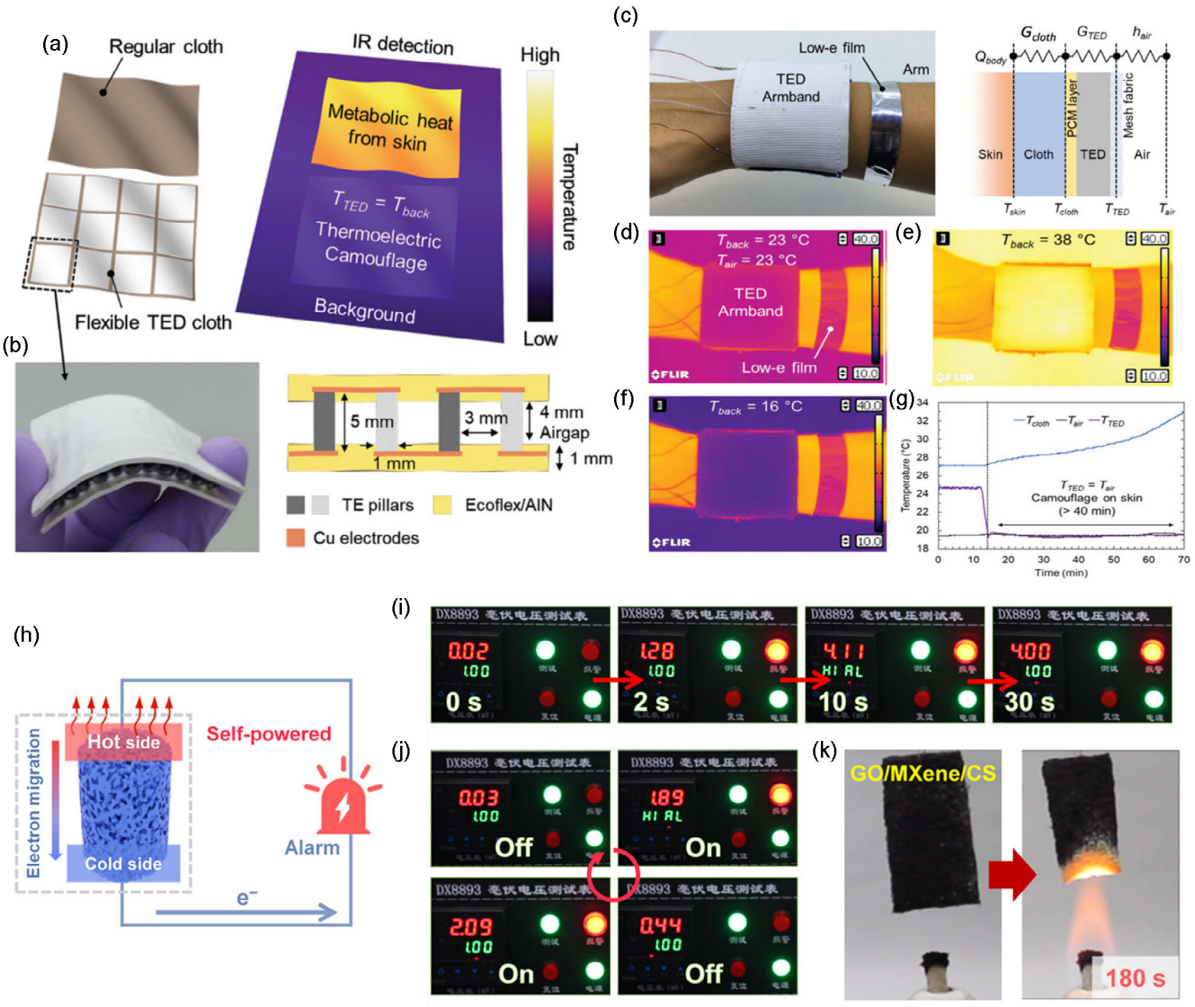
Emerging applications of w‐TEDs. (a, b) Conceptual illustration of thermal camouflage using flexible TED and photograph of the device. (c) Photograph of TED armband and low‐e Al strip attached to skin, along with its thermal resistance circuit. (d–f) IR images of TED armband and low‐e Al strip on human wrist at background temperatures of 23, 38, and 16 °C. (g) Long‐term camouflage performance of TED integrated with a PCM layer. Reproduced with permission [127]. Copyright 2020, Wiley‐VCH Verlag GmbH. (h) Schematic illustration of self‐powered fire‐warning test using an aerogel demonstrating thermoelectric property. (i) Operation of the fire warning system for 30 s under fire exposure and (j) reversible fire‐warning process of the aerogel. (k) Flame‐durable aerogel composed of graphene oxide (GO)/MXene/chitosan (CS). Reproduced with permission [128]. Copyright 2024, ACS Publications.

These studies advance thermal camouflage from passive concealment toward active control of the surface temperature and spatial thermal patterns. However, current systems mainly operate under predefined background conditions or programmed patterns. Practical camouflage will require environmental sensing and closed‐loop control that continuously adjust individual thermoelectric regions as the wearer moves and the surrounding thermal field changes. Extending this capability over large and deformable areas will also require careful management of power consumption and heat dissipation without adding excessive battery weight or bulky thermal management components. Validation under dynamic conditions will therefore be essential for translating controlled demonstrations into autonomous wearable camouflage systems.

Another potential application of thermoelectric materials is in fire‐hazard environments. He et al. developed a heat‐resistant self‐powered fire alarm system based on a graphene oxide (GO)/MXene/chitosan aerogel (Figure 15h) [128]. The fabricated aerogel exhibited an extremely fast thermal response, enabling fire detection within 1 s and sustaining fire warning for over 120 s in an externally powered conductivity‐based configuration owing to the rapid formation of conductive pathways and its excellent flame retardancy. When exposed to heat, the thermoelectric effect of MXene generates a potential difference that directly activates an alarm lamp without the need for an external power source. Specifically, the aerogel produced a voltage of 1.28 mV within 2 s, exceeding the threshold voltage required to trigger the fire alarm and maintaining a stable output for approximately 30 s (Figure 15i). Furthermore, the device consistently generated voltages above the threshold during successive flame‐on and flame‐off cycles, demonstrating reliable and rapid response characteristics (Figure 15j). In addition, the presence of MXene imparts excellent flame resistance to the aerogel, allowing it to maintain more than 80% of its original weight even after 180 s of direct flame exposure (Figure 15k). For practical fire warning, rapid thermoelectric response should be accompanied by reliable discrimination between hazardous heating and benign temperature fluctuations. System‐level validation should also confirm stable alarm thresholds and sufficient electrical output for audible or wireless notification before direct flame exposure.

### 5.4 Evaluation Indicators for Practical Wearability

Because w‐TEDs are designed to be worn directly on the human body and to continuously interact with the skin and physiological environment, their practical suitability cannot be determined solely by thermoelectric performance. In addition to this conventional performance metric, criteria related to the skin‐device interface, including wearer comfort, biosafety**,** and long‐term reliability, should be systematically considered to ensure safe and sustainable on‐body operation. Therefore, Table 3 summarizes the principal evaluation indicators related to these three aspects of practical wearability.

**TABLE 3:** Key indicators of comfort, biosafety, and long‐term reliability in w‐TEDs.

Category	Indicator	Key metric	Standard
Comfort	Air permeability	Air permeability (mm s^−1^ or L m^−2^ s^−1^)	ISO 9237
	Water vapor transmission rate (WVTR)	WVTR (g m^−2^ day^−1^)	ISO 2528, ISO 15496, ISO 15106
	Device thickness	Thickness (mm)	—
	Areal weight	Areal weight (mg cm^−2^)	—
	Bending stiffness	Bending stiffness (N m^2^)	—
	Thermal comfort	Skin temperature (°C), RH at the skin‐device interface (%), PMV, PPD (%), subjective thermal evaluation	ISO 7730:2025
	Skin adhesion and removability	Peel strength (N cm^−1^), residue	ISO 29862
Biosafety	Cytotoxicity	Cell viability (%)	ISO 10993‐5
	Skin irritation and sensitization	Irritation score, sensitization response	ISO 10993‐23, ISO 10993‐10
	Material leaching	Released ion or chemical concentration (mg L^−1^ or mg cm^−2^)	ISO 10993‐18
	Encapsulation integrity	WVTR (g m^−2^ day^−1^), leakage current (A), crack number density (mm^−1^ or mm^−2^), material‐leakage incidence (%)	ISO 2528, ISO 15106, IEC 60068‐2‐78, IEC TR 62899‐550‐1
Long‐term reliability	Sweat stability	Adhesion retention (%), output retention (%), encapsulation leakage or delamination, resistance change (%)	ISO 105‐E04
	Humid‐heat stability		IEC 60068‐2‐78
	Washing durability		ISO 6330
	Mechanical cycling	Cycle number, strain (%), bending radius (mm), resistance change (%), output retention (%)	—
	Prolonged on‐body operation	Operation time (h), voltage change (V), temperature change (°C), adhesion retention (%)	—

*Note:* PMV, predicted mean vote, represents the predicted average thermal sensation of a group; PPD, predicted percentage dissatisfied, represents the predicted proportion of individuals dissatisfied with the thermal environment.

A thick, heavy, or mechanically stiff device may fail to follow the skin curvature and motion, resulting in partial detachment, pressure concentration, or unstable contact. Limited air and water vapor permeability can increase temperature and humidity at the skin‐device interface, promoting discomfort, skin maceration, and adhesion loss during prolonged wear [130]. Therefore, skin adhesion must be sufficient to maintain conformal contact during movement, while removability must be controlled to minimize pain, residue, and skin damage. Accordingly, the practical evaluation of w‐TEDs needs to consider both subjective thermal perception and the mechanical stability of the skin‐device interface.

Biosafety and long‐term reliability are closely linked because the biological safety of a device may be affected by degradation during use [131]. Repeated stretching and bending, sweat and humidity exposure, thermal operation, and attachment‐detachment can cause microcracking of the encapsulation layer, electrode corrosion, interfacial delamination, and degradation of adhesive layers. These changes may create pathways through which thermoelectric constituents, metal ions, LMs, residual chemicals, or degradation products become exposed to the skin. Commonly used ITE materials based on Te, Sb, or Se can exhibit toxicity depending on their chemical form, dose, and route of exposure, potentially posing risks to both biological systems and the environment. Therefore, their biological and environmental safety should be evaluated, and appropriate end‐of‐life collection and disposal, together with recovery or recycling, where feasible, should be considered to minimize their release into the environment. More broadly, biosafety assessment should extend beyond one‐time testing of pristine devices and verify whether material isolation and electrical and thermal function remain intact after representative mechanical and environmental aging. From this perspective, long‐term reliability is not limited to performance retention but also contributes directly to maintaining biosafety throughout the intended service life.

More stringent validation is required for w‐TEDs intended for wounds or compromised skin. Unlike the epidermis, wounded tissue has a disrupted barrier and is exposed to exudate, variable pH [132], and an elevated risk of infection. These conditions may accelerate the degradation of adhesives and encapsulation layers and increase direct exposure of viable tissue to leachates or degradation products. Device removal may also damage newly formed tissue or increase the contamination risk [133]. Therefore, wound‐oriented w‐TEDs should be assessed not only for cytotoxicity and irritation but also for stability in exudative environments, microbial protection, atraumatic removal, and compatibility with tissue repair. For therapeutic systems, validation should demonstrate a clinically meaningful benefit without increasing the risks of infection, inflammation, or delayed healing.

Long‐term human wear or wound application studies should address not only technical efficacy but also participant protection and responsible data governance [134]. Such studies should be conducted after appropriate preclinical validation and should include independent ethical review, informed consent, risk–benefit‐based exposure durations, predefined stopping criteria, and follow‐up after device removal. Additional safeguards may be required for participants with chronic wounds or impaired healing because they may be more susceptible to adverse effects. When w‐TEDs continuously collect or wirelessly transmit physiological information, the types of data collected, storage duration, access rights, security measures, secondary use, and handling of data after participant withdrawal should also be defined in advance. Therefore, human validation should assess not only device safety and efficacy but also participant autonomy, privacy, and responsible management of physiological data.

## 6 Design Considerations and Future Directions for Wearable Thermoelectric Devices

Throughout this review, we have examined w‐TEDs developed for energy harvesting, thermal regulation, sensing, and other emerging applications, together with the material and structural strategies used to improve their thermoelectric performance and wearability. Because the reported voltage and power depend strongly on the applied temperature difference, device area, and number of thermoelectric pairs, raw output values alone do not adequately reveal the effects of these design strategies. Table 4 therefore compares representative devices using normalized electrical metrics where applicable while also considering their thermal regulation capability, mechanical adaptability, and measurement environment. The results highlight the high performance already achieved in the field. TEGs have reached normalized power density values of up to 25.34 μW cm^−2^ K^−2^, and TECs have produced temperature modulation approaching 20 K under controlled conditions. However, these values are obtained from devices designed for different functions and evaluated under different thermal boundary conditions. Their mechanical requirements also vary from simple bending to large tensile deformation. The table should therefore be interpreted as a comparison of achievable performance ranges and design trade‐offs rather than as a direct ranking of individual devices.

**TABLE 4:** Summary of representative w‐TEDs discussed in this review.

Mode	p‐type	n‐type	*S*, μV K^−1^	Wearability strategy	*P*_norm_, μW cm^−2^ K^−2^	*V*_norm_, mV K^−1^ pair^−1^	Thermal regulation performance	Response time	Deformation	Testing environment	References
TEG	Sb_2_Te_3_	Bi_2_Te_3_	p‐type: 124 n‐type: 150	Rigid thermoelectric cuboids embedded in Ecoflex with serpentine electrodes	0.386	0.1188	—	—	Max <80% strain 50% strain, 100 cycles	Complex and dynamic curved surfaces	[32]
TEG/TEC	Bi‐Te based materials		—	Serpentine wiring with asymmetric encapsulation; top exposed, skin side sealed	0.006	0.0668	Cooling Δ*T*_max_ = 5 °C at 0.5 V	—	Max <30.6% strain 30% strain, 1000 cycles	2 m s^−1^ airflow for TEG	[33]
TEG	Bi_0.3_Sb_1.7_Te_3_	Bi_2_Te_3_ + Ru	p‐type: 152.5 n‐type: 157.5	Laser‐cut pop‐up kirigami architecture	0.242	0.1538	—	—	Max <70% strain 40% strain, 1000 cycles	Curved cylindrical heat source	[34]
TEG	Bi_2_Te_3_	Bi_2_Te_3_	—	Liquid‐metal‐embedded elastomer as interconnect and PDMS filler	0.024	0.0928	—	—	Max >50% strain 30% strain, 1000 cycles	Bench‐top thermal gradient	[35]
TEG	Bi_0.5_Sb_1.5_Te_3_	Bi_2_Se_0.3_Te_2.7_	p‐type: 220 n‐type: 220	Liquid‐metal interconnects with low‐k silicone composite filler	0.714	0.1902	—	—	Bending (*r* = 6 mm), 100 cycles	1.2 m s^−1^ airflow for TEG	[36]
TEG/TEC	Bi_2_Te_3_	Bi_2_Te_3_	p‐type: 200–235 n‐type: 200–235	3D soft architecture with 12% infill hollow‐microsphere elastomer	1.154	0.8093	Cooling Δ*T*_max_ = 11 °C at 1 A	—	Max <230% strain 50% strain, 2000 cycles	Bench‐top thermal gradient	[37]
TEG	Bi_0.5_Sb_1.5_Te_3_	Bi_2_Te_2.7_Se_0.3_	—	Sputtered thin films, Ti interfacial layers, flip‐chip bonding	4.35	0.3784	—	—	Bending (*r* = 10 mm), 100 cycles	Bench‐top thermal gradient	[47]
TEG	Cu_2_Se	Ag_2_Se	p‐type: 17.5–23.0 n‐type: 84.2–134.0	Double‐sided in‐situ selenized films on PI; Ag‐paste interconnection	0.065	0.0437	—	—	Bending (*r* = 2–6 cm), 1500 cycles	Bench‐top thermal gradient	[48]
TEG	—	Ag_2_Se	n‐type: 130	Ag_2_Se synthesized on melamine‐foam 3D template	0.0154	0.0538	—	—	Max >100% strain bending (*r* = 5 mm), 200 cycles	On‐skin measurement	[49]
TEG	PEDOT:PSS‐coated CNTF	Oleamine‐doped CNTF	n‐type: 64	1D woven thermoelectric fibers	0.0035	—	—	—	Max <80% strain bending (91°), 100 cycles	Bench‐top thermal gradient	[75]
TEG	PEDOT:PSS cotton	PEI‐treated CNT fabric	p‐type: 17 n‐type: 58	Dipped functional textile pairs	—	0.0941	—	—	—	Bench‐top thermal gradient	[78]
TEG	SWCNT doped with PAA	SWCNT doped with PEI	—	Out‐of‐plane 3D‐compliant structure with PDMS substrate/encapsulation	0.000124	0.0746	—	<Tens of milliseconds	Bending (*r* = 23.12 mm (*x*)/13.51 mm (*y*)), 1000 cycles 5% strain, 1000 cycles	On‐skin measurement Accelerated stress test at 85 °C/85% RH	[76]
TEG	CNT/PEDOT:PSS	CNT/oleylamine	p‐type: ~52 n‐type: ~42	3D warp‐knitted spacer fabric with CNT	—	0.085	—	Temperature response 240 ms, strain/compression response 20 ms	50% compressive strain, >10 000 cycles	Bench‐top thermal gradient	[77]
TEG	SWCNT + PEDOT:PSS composite	—	p‐type: 51.29	PET core‐sheath fibers, porous breathable 3D fabric	—	—	—	Compression response 20 ms	Pressure 15 kPa, 6000 cycles 6 h wash test	Bench‐top thermal gradient	[79]
TEG/TEC	Bi_2_Te_3_	Bi_2_Te_3_	p‐type: 223.43 n‐type: 228.85	Semi‐liquid‐metal interconnectors, graphene/PU encapsulation and flexible paraffin PCM heat sink	0.217	0.4834	Cooling Δ*T*_max_ = ~10 °C at ambient 24 and 35 °C	—	Max 15% strain bending (*r* = 20 mm), 1000 cycles	On‐simulated skin measurement	[90]
TEG/TEC	Bi_0.5_Sb_1.5_Te_3_	Bi_2_Te_2.7_Se_0.3_	p‐type: 174.3 n‐type: 191.5	Legs embedded in disulfide‐crosslinked polyurethane (DSPU), liquid‐metal electrodes encapsulated with DSPU	3.14	0.172	Cooling Δ*T*_max_ = 6.2 K at 0.8 A (two‐layer tower); 3.9 K single‐layer	—	Max 25% strain 10% strain, 2000 cycles	Bench‐top thermal gradient	[92]
TEG	Sb_1.5_Bi_0.5_Te_3_	Bi_2_Se_0.2_Te_2.8_	p‐type: 204.98 n‐type: 203.06	Flexible PI substrate with Cu electrodes	25.34	0.343	—	—	—	Forced water cooling	[93]
TEG	Bi_2_Te_3_	Bi_2_Te_3_	—	—	0.267	—	—	—	—	Under vacuum, at fixed heat flux	[96]
TEG	Ni_90_Cr_10_	Cu_55_Ni_45_ or Ni_95_Al_2_Mn_2_Si_1_	p‐type: <20	—	0.183	0.0742	—	—	—	Vacuum chamber with a hot/cold‐plate gradient	[94]
TEC	Commercial TEC1‐12706, TEC2‐15804		Device level: 38 800	—	—	—	Cooling *T*_min_ = −64.3 °C at 12 V	—	—	Water‐cooled two‐stage TEC	[97]
TEC	Bi_2_Te_3_	Bi_2_Te_3_	—	Island‐bridge structure; thermoelectric unit as an island connected by serpentine interconnects and partially embedded in elastomeric material sheets	—	—	11 °C on‐skin temperature reduction from 32–21 °C	—	Max <20% strain 10% strain, 350 cycles	On‐skin measurement with driving‐power regulation	[95]
TEG	Bi_0.5_Sb_1.5_Te_3_	Bi_2_Te_2.7_Se_0.3_	p‐type: ~280 n‐type: ~150	—	0.082	—	—	—	—	Bench‐top thermal gradient	[104]
TEG	BiSbTe	BiTeSe	—	—	9.64	—	—	—	—	Bench‐top thermal gradient	[105]
TEG	Bi_2_Te_3_	Bi_2_Te_3_	—	Rigid thermoelectric legs bonded to elastic conductors via EMGL; fully PDMS‐encapsulated	1.48	0.235	—	—	Max <20% strain (device)	On‐forearm measurement during cold‐water immersion	[106]
TEG	—	CND/PVP	n‐type: 358.45	Flexible CND/PVP thin films drop‐cast on biocompatible cellulose substrate	—	—	—	—	*r* = 7.5 mm bending	On‐skin measurement	[107]
TEG	PEDOT:PSS	—	p‐type: 15	Inkjet‐printed Ag interconnects on flexible PEN substrate	0.00000136	0.01275	—	—	—	Bench‐top thermal gradient	[109]
TEG/TEC	Bi‐Te based materials		—	Rigid thermoelectric legs on flexible FPCB with liquid‐metal skeleton elastomer	3.613	—	Cooling Δ*T*_max_ = 20 °C (324 W/m^2^)	—	Max >600% strain (matrix) bending (*r* = 35 mm), 1200 cycles	Curved surface with water flow	[110]
TEG	Bi_2_Te_3_	Bi_2_Te_3_	p‐type: 147.2 n‐type: 164.8	AgNW soft electrodes with magnetically self‐assembled Ag‐Ni/PDMS heat conductors	0.262	0.24	—	—	Max 20% strain 10% strain, 600 cycles	On‐skin measurement	[112]
TEG	Bi_2_Te_3_	Bi_2_Te_3_	—	Low‐stress Cu electrode array on PI substrate	0.85	0.233	—	—	Bending (*r* = 30 mm)	On‐skin measurement	[113]
TEG	CNT	—	p‐type: 49	Knitted fabric with CNTs and electrosprayed TPU microspheres	—	—	—	Temperature response 0.9 s, strain/compression response 0.3 s	Max 250% strain bending 4000 cycles/ stretching 1000 cycles washing 1000 cycles	High‐humidity and artificial‐sweat conditions Facial‐mask breathing test	[114]
TEG	rGO/rPEDOT:PSS	—	p‐type: 29.5 ± 1.7	Flexible thin film	—	—	—	—	Bending (45° and 90°), 1000 cycles	Hot‐object contact on a nitrile glove with sensors	[115]
TEG	Bi_0.5_Sb_1.5_Te_3_	Bi_2_Te_3_	p‐type: ~260 (at 360 K) n‐type: ~180 (at 360 K)	Flexible thin film on PI substrate	—	0.1667	—	—	Bending (*r* = 12 mm), 400 cycles	Bench‐top thermal gradient	[120]
TEG	Single ionic thermoelectric material TA‐loaded poly(AM‐co‐AMPS‐Na)		12 150	Adhesive and highly stretchable hydrogel with flexible textile electrode	—	—	—	—	Max >250% strain bending, twisting, and folding	Bench‐top thermal gradient	[121]
TEG	Bi_2_Te_3_	Bi_2_Te_3_	4440 (device‐level)	Rigid Bi_2_Te_3_ legs embedded in a flexible Ecoflex matrix	—	0.0888	—	—	Bending (*r* = 10–40 mm), 1000 cycles	Bench‐top thermal gradient	[123]
TEG	Cotton/PEDOT:PSS/DMSO/GOPS yarn	Constantan wire	p‐type: ~19	All‐fiber woven integration for conformability, stretchability, and breathability	0.00239	—	—	—	Bending (90°), 1000 cycles	Bench‐top thermal gradient	[124]
TEC	Bi_2_Te_3_/Sb_2_Te_3_ superlattice	Bi_2_Te_3_/Bi_2_Te_2_._83_Se_0_._17_ superlattice	p‐type: 230 n‐type: 230	Thin‐film architecture	—	—	Cooling by 10–20 °C below ambient	~3 s	Skin‐mounted use	Human thermal‐perception experiments	[125]
TEC	Bi‐Te based p‐ and n‐type thermoelectric pillars		196 μV K^−1^ per pillar (average value at the device‐level)	Rigid thermoelectric cuboids embedded in Ecoflex with serpentine electrodesand combined with a skin‐side PCM/elastomer layer for thermal comfort	—	—	>±20 °C relative to simulated skin on‐body camouflage from 16 to 38 °C	<10 s below 35 °C <20 s at higher background temperatures	Bending (*r* = 2 cm)	On‐body IR‐camera test	[127]
TEC	Bi_0.5_Sb_1.5_Te_3_	Bi_2_Te_2.7_Se_0.3_	—	Kirigami‐patterned Cu interconnects on a PI‐based FPCB	—	—	5.77 °C minimum and 109.76 °C maximum at 1.9 A	2.71 s	Curved and on‐arm bending demonstrated	Under ambient‐air conditions Operating temperature range: 5.77–109.16 °C	[129]
TEG	GO/MXene/chitosan aerogel		—	Free‐standing lightweight porous aerogel	—	—	—	• Externally powered mode: ‐ fire detection within 1 s ‐ warning duration >120 s. • Self‐powered thermoelectric mode: ‐ 1.28 mV generated within 2 s ‐ stable output for approximately 30 s	Max ~50% compressive strain	Direct flame exposure	[128]
TEG	Bi_0.5_Sb_1.5_Te_3_	Bi_2_Te_3_	p‐type: 70–85 n‐type: 115–120	Inkjet‐printed ultrathin printed thermoelectric film (<0.89 μm) with LM interconnects on PI	—	0.3	—	—	Bending (*r* = 5.5 mm), 50 cycles	Curved plastic cup with warm water (30 °C)	[40]
TEG	Bi_0.4_Sb_1.6_Te_3_	Bi_2_Te2.7Se_0.3_	p‐type: 170–190 n‐type: 114–134	Brush‐paintable thermoelectric ink for shape‐conformable deposition	0.181	0.2035	—	—	—	Hemispherical alumina substrate Bench‐top thermal gradient	[41]
TEG	PEDOT:PSS	Ag_2_Se	p‐type: 25.4 n‐type: 220	Screen‐printable thermoelectric inks with lightweight PEN architecture	0.00733	0.1823	—	—	—	Bench‐top thermal gradient	[42]
TEG	Bi_0.4_Sb_1.6_Te_3_ + 5 wt% Te	Bi_2_Te_3_ + 7.5 wt% Te	p‐type: ~190 n‐type: ~190 (at 303 K)	Screen‐printable thermoelectric inks with thermoelectric nanobinders on PI	3	0.0863	—	—	Bending (*r* = 8 mm), 1000 cycles	Bench‐top thermal gradient	[43]
TEG	(0.25 Bi, 0.75 Sb)_2_(0.95Te,0.05Se)_3_	(0.98 Bi, 0.02 Sb)_2_(0.9Te,0.1Se)_3_	p‐type: ~115 n‐type: ~260	Dispenser‐printable thermoelectric pastes integrated into a flexible polyester fabric	—	0.0885	—	—	—	Bench‐top thermal gradient	[44]
TEG	—	Bi_2_Te_2.7_Se_0.3_	163.4	Aerosol‐jet‐printable thermoelectric ink for 3D conformal printing	1.08	—		—	Bending (*r* = 1.5–10 mm), 500 cycles	Bench‐top thermal gradient	[45]
TEG	Bi_0.5_Sb_1.5_Te_3_	Bi_2_Te_2.7_Se_0.3_	p‐type: 219 n‐type: 176	Tailorable and foldable Bi_2_Te_3_/cellulose thermoelectric paper	0.00826	0.803	—	—	5 mm displacement 3000 bending cycles	Bench‐top thermal gradient	[46]

*Note:* *P*_norm_ = *P*_density_/Δ*T* ^2^ and *V*_norm _= *V*_OC_/(*N* Δ*T*), where *N* is the number of thermocouple pairs, and Δ*T* is the temperature difference across the device. All Seebeck coefficient (*S*) values are presented as absolute values for consistency; accordingly, the negative signs for n‐type materials are omitted.

Figure 16 translates these trade‐offs into an application‐oriented design framework that begins with the intended operating mode. A TEG must generate useful voltage and power from the limited heat flux and temperature difference available on the body, whereas a TEC must provide controlled heating or cooling while dissipating both transported heat and internally generated Joule heat (Figure 16a). Once this functional requirement is established, the target body site defines the mechanical boundary conditions of the device (Figure 16b). Relatively flat regions impose mainly bending and conformability requirements, while joints and other highly mobile regions generate larger, localized, and dynamically varying strains. Device size further influences these conditions. A compact device near a joint may experience concentrated deformation, whereas a large‐area device may span multiple curvatures and exhibit spatially nonuniform strain and skin contact. These mechanical demands, in turn, determine the appropriate wearable design strategy (Figure 16c). When deformation is modest, high‐performance inorganic thermoelectric elements integrated on an FPCB‐type platform can provide sufficient bending compliance without substantially compromising the thermoelectric performance. As greater conformity and deformation tolerance are required, stretchable substrates and interconnects become necessary to isolate strain from the rigid thermoelectric elements. Intrinsically deformable thermoelectric materials become advantageous when structural designs alone can no longer accommodate the required deformation, although this transition commonly reduces the thermoelectric fill factor and introduces additional electrical and thermal losses. The device architecture should therefore be selected according to the level of mechanical adaptation required while preserving a sufficient active area, electrical conduction, and heat transfer. Within the selected architecture, performance enhancement requires coordinated control of electrical, thermal, and interfacial losses (Figure 16d). Low‐resistance interconnects and stable contacts help preserve the generated voltage and reduce Joule heating, while optimization of the thermal pathway and heat sink sustains the temperature gradient required for power generation or cooling. Conformal skin contact supports both functions by reducing interfacial thermal resistance and minimizing local air gaps. Because these factors are strongly coupled, improvements in individual components should ultimately be verified through complete device measurements rather than evaluated in isolation.

**FIGURE 16:**
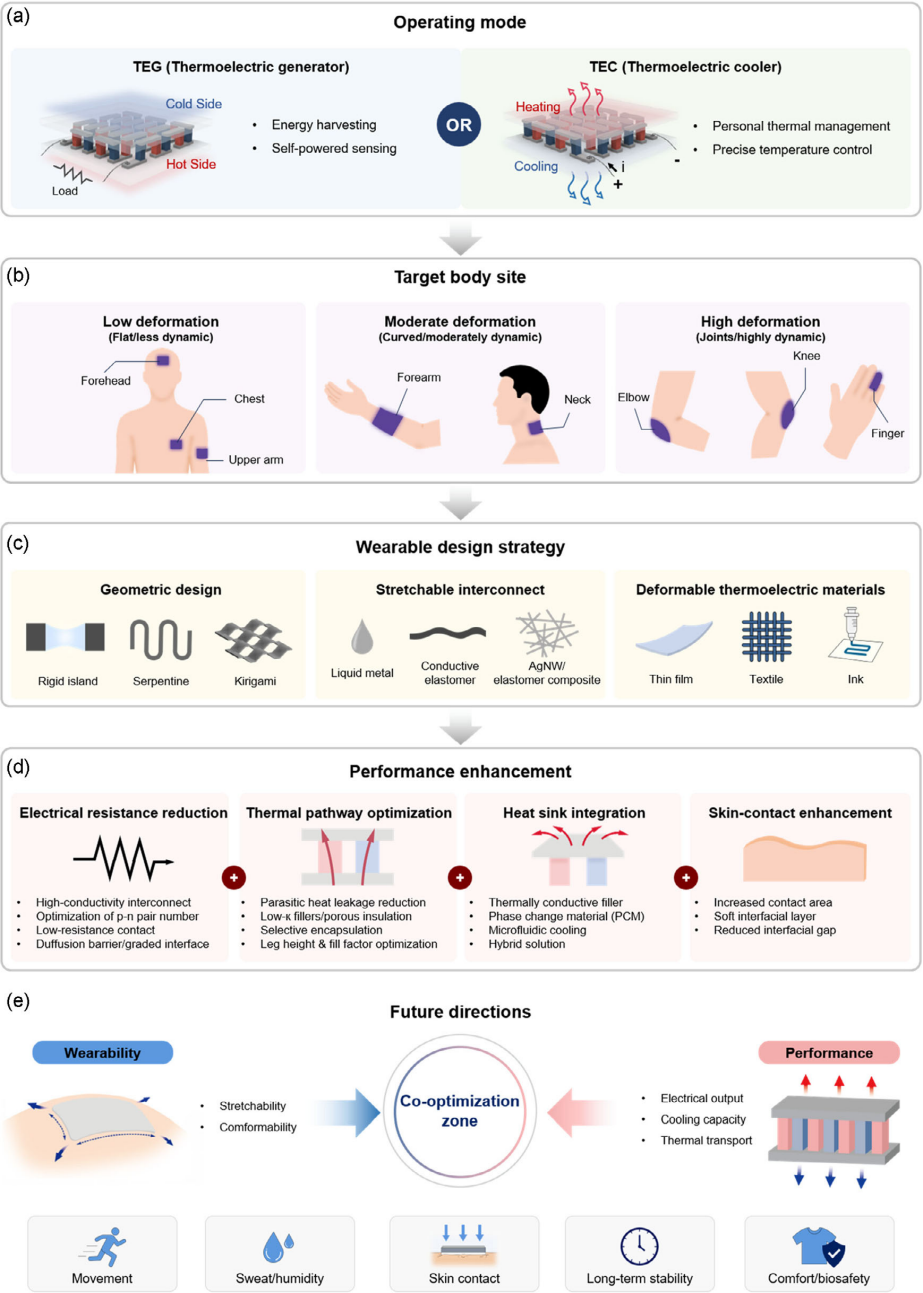
Design framework for the fabrication and optimization of w‐TEDs. (a) Selection of the operating mode as either a TEG or a TEC. (b) Classification of the target body site and corresponding deformation requirements, ranging from low deformation on relatively flat regions to high deformation at dynamic joints. (c) Selection of an appropriate wearability strategy, including geometry modification, stretchable interconnect, and deformable thermoelectric materials. (d) Device‐level performance optimization through electrical resistance reduction, thermal pathway optimization, heat sink integration, and skin contact enhancement. (e) Co‐optimization of wearability and thermoelectric performance, followed by validation under realistic conditions involving motion, sweat, and humidity, variable skin contact, long‐term operation, and wearer comfort and biosafety.

Future development should therefore focus on expanding the co‐optimization zone between wearability and thermoelectric performance rather than maximizing either property independently (Figure 16e). For highly deformable TEGs, a normalized power density on the order of 1 μW cm^−2^ K^−2^ may provide a practical system‐level benchmark based on the performance range summarized in Table 4. For TECs, maintaining a temperature modulation of approximately 10 K would represent a similarly meaningful target. More importantly, these values should be retained during site‐relevant cyclic deformation and under realistic thermal boundary conditions rather than be demonstrated separately from maximum bending or stretchability. For large‐area systems, performance should also remain spatially uniform despite variations in curvature and skin contact across the device. To determine whether these co‐optimized performance targets can be sustained in practice, evaluation should move beyond isolated maximum‐performance tests toward combined conditions that reflect actual wear, including cyclic deformation, sweat and humidity exposure, variations in skin contact, long‐term operation, and wearer comfort and safety. These factors act simultaneously during wear and can collectively affect electrical stability, heat transfer, mechanical integrity, and skin compatibility. Demonstrating stable complete‐device operation under such coupled conditions would provide a more meaningful indication of practical wearability than further increasing an isolated record value under controlled laboratory conditions.

## 7 Conclusion

Accelerating climate change and the growing energy footprint of modern technologies have intensified the demand for sustainable solutions that combine efficient energy utilization with precise thermal regulation. In this context, w‐TEDs, which leverage the ubiquitous temperature difference between the human body and the surrounding environment, have emerged as promising candidates for both PTM and energy harvesting. However, commercially available conventional TED modules are typically bulky and rigid, hindering conformal contact with the curved surface of the human body and causing wearer discomfort. Such poor conformability also reduces effective heat transfer, thereby limiting the overall device performance.

To address these challenges, this review presented design strategies for developing w‐TEDs with improved wearability and device performance. We discussed three major approaches for enhancing wearability, focusing on geometric design and mechanical adaptation, and outlined key strategies for improving device performance through electrical and thermal design considerations. We also highlighted representative applications of w‐TEDs and discussed emerging opportunities for their broader implementation across diverse technological fields.

Despite significant progress in the development of w‐TEDs, a fundamental challenge persists: the inherent trade‐off between the thermoelectric performance and mechanical compliance. Materials and structural designs used to impart stretchability often increase device‐level electrical resistance and introduce parasitic thermal pathways, thereby reducing the overall device efficiency and weakening the effective temperature gradient across the thermoelectric elements. In addition, the practical realization of w‐TEDs requires the assembly of arrays of thermoelectric legs together with deformable substrates, stretchable interconnects, and integrated thermal management and packaging components. Integrating these heterogeneous elements into a thin, compliant, and wearable form factor remains highly challenging because conventional fabrication and packaging strategies developed for rigid TEDs are not readily compatible with soft platforms. As a result, most w‐TEDs remain at the laboratory scale, and scalable manufacturing of large‐area, ultrathin devices is still difficult.

To enable practical deployment, next‐generation w‐TEDs should be capable of conformally covering relatively large and curved body surfaces, such as the back of the neck, while remaining mechanically stable under body motion. At the same time, they should deliver meaningful device‐level performance, including temperature modulation exceeding 10 K or power generation above 100 μW cm^−2^ at Δ*T* ≈ 10 K, depending on the operating mode. Achieving these goals will require deformable materials with high thermoelectric performance, along with optimized device design to preserve efficiency under wearable operating conditions. It will also require the efficient integration of diverse functional components without sacrificing mechanical compliance or operational stability. Ultimately, such integration must be enabled by simple and scalable manufacturing strategies, rather than labor‐intensive assembly, to support the large‐area production of practical w‐TEDs. Continued progress in these areas will help transform w‐TEDs from standalone power generators and thermal regulators into comprehensive bioelectronic platforms for clinical monitoring, human–machine interfacing, AI‐assisted adaptive control, and autonomous self‐powered sensing. In this way, w‐TEDs may ultimately bridge the gap between laboratory prototypes and real‐world wearable systems.

## Funding

This work was supported by the National Research Foundation of Korea (RS‐2025‐22732971 and RS‐2025‐18362970), the Ministry of Education (2025‐RISE‐01‐022‐02), and the Seoul Metropolitan Government (2025‐RISE‐01‐022‐02 and VC250026).

## Conflicts of Interest

The authors declare no conflicts of interest.

## Data Availability

Data sharing is not applicable to this article as no datasets were generated or analyzed during the current study.
